# Gene loss and symbiont switching during adaptation to the deep sea in a globally distributed symbiosis

**DOI:** 10.1038/s41396-022-01355-z

**Published:** 2023-01-13

**Authors:** Jay T. Osvatic, Benedict Yuen, Martin Kunert, Laetitia Wilkins, Bela Hausmann, Peter Girguis, Kennet Lundin, John Taylor, Guillaume Jospin, Jillian M. Petersen

**Affiliations:** 1 University of Vienna, Centre for Microbiology and Environmental Systems Science, Department for Microbiology and Ecosystem Science, Division of Microbial Ecology, Djerassiplatz 1, 1030 Vienna, Austria; 2 University of Venna, Doctoral School in Microbiology and Environmental Science, Djerassiplatz 1, 1030 Vienna, Austria; 3 Eco-Evolutionary Interactions Group, Max Planck Institute for Marine Microbiology, Celsiusstrasse 1, 28209 Bremen, Germany; 4 Joint Microbiome Facility of the Medical University of Vienna and the University of Vienna, 1030 Vienna, Austria; 5 Department of Laboratory Medicine, Medical University of Vienna, 1090 Vienna, Austria; 6 Department of Organismic and Evolutionary Biology, Harvard University, Cambridge, MA 02138 USA; 7 Gothenburg Natural History Museum, Box 7283, 40235 Gothenburg, Sweden; 8 Gothenburg Global Biodiversity Centre, Box 461, 40530 Gothenburg, Sweden; 9 Natural History Museum, Cromwell Rd, London, SW7 5BD UK; 10 AnimalBiome, 400 29th Street, Suite 502, Oakland, CA 94609 USA

**Keywords:** Biogeography, Symbiosis, Microbial ecology, Environmental microbiology

## Abstract

Chemosynthetic symbioses between bacteria and invertebrates occur worldwide from coastal sediments to the deep sea. Most host groups are restricted to either shallow or deep waters. In contrast, Lucinidae, the most species-rich family of chemosymbiotic invertebrates, has both shallow- and deep-sea representatives. Multiple lucinid species have independently colonized the deep sea, which provides a unique framework for understanding the role microbial symbionts play in evolutionary transitions between shallow and deep waters. Lucinids acquire their symbionts from their surroundings during early development, which may allow them to flexibly acquire symbionts that are adapted to local environments. Via metagenomic analyses of museum and other samples collected over decades, we investigated the biodiversity and metabolic capabilities of the symbionts of 22 mostly deep-water lucinid species. We aimed to test the theory that the symbiont played a role in adaptation to life in deep-sea habitats. We identified 16 symbiont species, mostly within the previously described genus *Ca*. Thiodiazotropha. Most genomic functions were shared by both shallow-water and deep-sea *Ca*. Thiodiazotropha, though nitrogen fixation was exclusive to shallow-water species. We discovered multiple cases of symbiont switching near deep-sea hydrothermal vents and cold seeps, where distantly related hosts convergently acquired novel symbionts from a different bacterial order. Finally, analyses of selection revealed consistently stronger purifying selection on symbiont genomes in two extreme habitats - hydrothermal vents and an oxygen-minimum zone. Our findings reveal that shifts in symbiont metabolic capability and, in some cases, acquisition of a novel symbiont accompanied adaptation of lucinids to challenging deep-sea habitats.

## Introduction

Numerous environments in Earth’s oceans are characterized by the availability of both molecular oxygen and reduced compounds, such as hydrogen sulfide, methane, and hydrogen; conditions where chemosynthetic organisms can thrive [1]. Diverse invertebrate lineages have evolved to exploit these niches by tapping into the metabolic capabilities of chemosynthetic bacteria through symbiotic associations that have allowed them to thrive in a diverse range of environments such as deep-sea hydrothermal vents and cold seeps, shallow coastal seagrass beds, and coral reefs [1–7]. Despite their geochemical similarities, these environments can vary greatly in the concentrations and availability of particular reduced compounds, oxygen and other nutrients that support chemosynthetic symbioses [8]. As a result of these differences, invertebrates have formed chemosymbiotic associations with numerous phylogenetically distinct lineages of sulfur-oxidizing chemolithoautotrophic bacteria that may be specialized for particular niches [6]. These bacteria are often acquired from the surrounding environment, a strategy allowing the host to preferentially select bacteria better adapted to local conditions, and in some cases, the host may switch symbionts and acquire novel, distantly related bacterial symbionts if these confer greater benefit [9]. Symbiont switching holds immense potential for evolutionary novelty, as the rapid acquisition of novel symbiont phenotypes could allow expansions into novel ecological niches, such as different habitats or trophic modes. However, novel symbiotic partner combinations can also be inefficient due to a lack of coevolutionary history and novel host-symbiont associations can have negative fitness consequences that might be exacerbated by novel environmental conditions [10]. Insights into the biodiversity and metabolic function of bacteria in these flexible relationships is critical to understanding how the environment shapes host-microbe symbioses.

The bivalve family Lucinidae is a species-rich group known mostly from shallow-waters. The highest diversity is typically found in seagrass and coral reef environments in the tropical Atlantic and Indo-West Pacific [11]. All lucinids studied to date form a symbiosis with chemolithoautotrophic Gammaproteobacteria [12–15], which they acquire from the environment during early development [16–18]. Recent genomic studies have unveiled the diversity of symbionts associated with shallow-water lucinids from the Caribbean and Mediterranean, as well as provided insights into their core metabolic functions, which include sulfur oxidation, carbon fixation, and nitrogen assimilation [12–15, 19]. Additionally, the ability to fix nitrogen has been found to be a hallmark trait of the *Ca*. Thiodiazotropha symbiont genus, the primary symbiont lineage associated with lucinid clams [12], and is likely to be a critical adaptation to life in oligotrophic and nitrogen-limited seagrass and coral reef environments [13, 20, 21]. Despite recent progress in understanding the global biogeography and genomic potential of the lucinid symbionts [12–15, 19], much of our existing knowledge is limited to studies in the Caribbean and Mediterranean, and these efforts only scratch at the surface of the diversity within the lucinid family, as species from temperate and deep-water habitats have often been overlooked [12–15, 19].

**Fig. 1:**
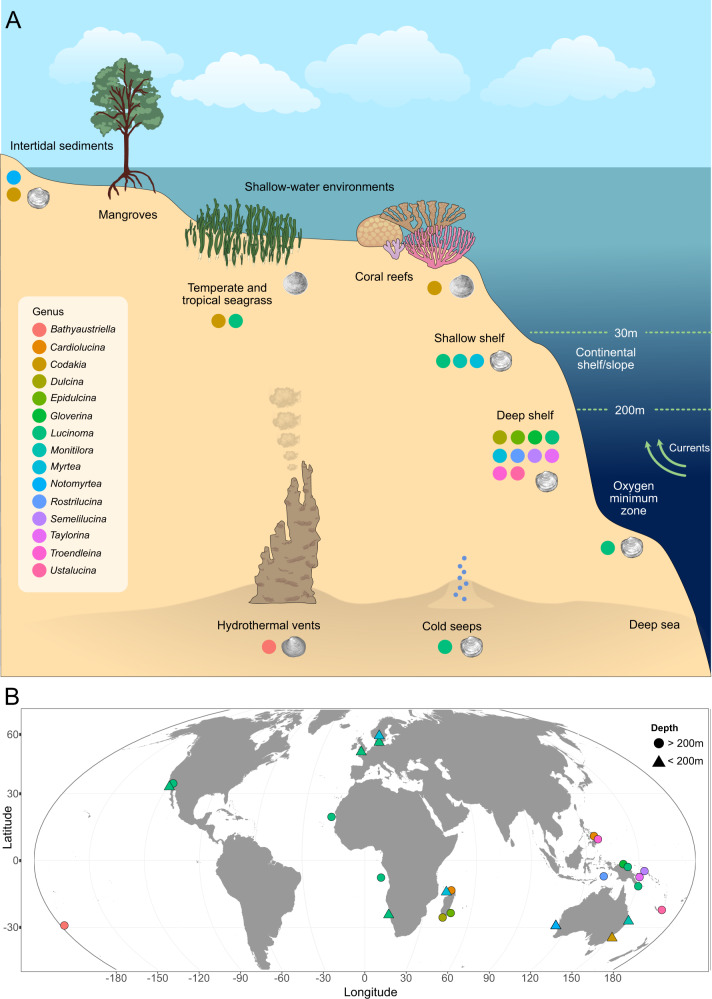
Diverse Lucinidae from a range of deep and shallow-water habitats around the world were investigated using metagenomics. **A** The lucinids sampled in this study, colored by genus, originated from diverse shallow- and deep-water habitats including intertidal, cold-seep, hydrothermal vent, and oxygen-minimum zone sediments. **B** Sampling locations of the lucinid species sequenced in this study, colored by genus. Shape of symbols indicate sampling depth (shallower or deeper than 200 m below the sea surface).

Deep-water sampling expeditions around the Indo-West Pacific and Madagascar over the last decade led to the discovery of an unexpected diversity of lucinids, many previously unknown to science, in offshore shelf and bathyal sediments (Fig. 1A) [22]. The evolutionary history of deep-water lucinids is diverse and complex. While some groups, such as members of the genus *Lucinoma*, have radiated primarily in deeper-water habitats around the globe, other taxa are derived from independent deep-water colonization events originating from shallow-water ancestors [23]. Deep-water lucinids have colonized environments where an abundant supply of reduced sulfur compounds, of either biogenic or geothermal origin, occurs in close proximity to oxidants like oxygen and nitrate (e.g. sediments enriched by sunken vegetation and organic falls, hydrocarbon seeps, and hydrothermal vents) [23–25]. The physical conditions in these deep-water environments pose very different challenges compared to tropical shallow-water coastal habitats such as low temperatures [26–28], intense pressure [26, 27, 29], and in some environments, limited oxygen availability [26]. These environments also have unique trophic structures compared with the photic zone where photosynthesis powers most primary production [26, 30]. These disparate ecological niches also differ in their biogeochemical conditions, particularly in the concentration of reduced sulfur compounds and other nutrients critical to chemosynthetic symbiosis [8]. However, we have scarce information on the diversity and metabolic capabilities of the sulfur-oxidizing symbionts of lucinids from deep-water habitats.

Herein we used high-throughput metagenomic sequencing to study the diversity and metabolic capabilities of symbionts associated with lucinids from a diverse range of deep-water environments around the world (Fig. 1). We considered depths greater than 200 m as deep water according to the definitions used by Cosel and Bouchet [31] and Glover and Taylor [22]. Our objectives were: (1) explore the phylogenetic relationships among shallow and deep-water symbiont taxa to investigate how the diversification of lucinids across a range of environments has shaped symbiont choice; (2) compare the metabolic capabilities of symbionts from deep versus shallow-water environments to understand how different environments have shaped the lucinid symbioses; (3) infer evidence for adaptive evolution in symbiont genomes by estimating the non-synonymous to synonymous substitution ratio of protein-coding genes.

Our findings reveal the presence of multiple previously undescribed *Ca*. Thiodiazotropha species found only in lucinids from habitats in deeper waters and/or higher latitudes. A single *Ca*. Thiodiazotropha species, *Ca*. T. gloverae, was found in a wide range of both deep sea and temperate shallow-water locales. Additionally, we report that four deep-water lucinid species, two of which inhabit sites where reduced compounds originate from geothermal sources, have switched from associating with symbionts from the *Ca*. Thiodiazotropha genus to associations with a distinct group of sulfur-oxidizing Gammaproteobacteria closely related to the symbionts of hydrothermal vent gastropods. These symbiont species share large portions of their core metabolic functions but vary in nitrogen metabolism, e.g., nitrogen fixation is absent in nearly all symbionts from deep and temperate waters. This study provides new insights into the diversity and biogeography of the lucinid symbionts and highlights how their metabolic capabilities and the flexibility of lucinid symbiosis has shaped the radiation of this species-rich bivalve family across a myriad of environments in the ocean.

## Methods

### Sample collection

#### Fresh clams

*Lucinoma aequizonata* [32] specimens were collected from anoxic sediments in the Santa Barbara Basin, off the coast of California, USA (2019) during an expedition FK181005 on the *RV Falkor*. Clams were collected using the remotely operated vehicle SUBastian with an attached sifter to remove sediments. Gills were dissected upon arrival at the water’s surface and stored in RNAlater (Cat. No. AM7020; Life Technologies, USA) and stored at 4 °C overnight and then frozen at −20 °C until extraction.

#### Museum samples

Gill tissue fragments were removed using sterile scalpels and forceps from samples obtained from the collections of the Natural History Museum (NHM) London, UK, the Muséum National d’Histoire Naturelle (MNHN), Paris, France, the Museum of Comparative Zoology, Harvard University, Boston, MA, USA, and the Göteborgs Naturhistoriska Museum, Sweden (GNM). All museum collection samples had been preserved in 90–100% ethanol and stored at 13–25 °C.

### DNA extraction and library preparation for metagenome and amplicon sequencing

DNA was extracted from the gill tissues of *Bathyaustriella thionipta* [25] *n* = 5, *Cardiolucina* cf. *quadrata* [33] *n* = 2, *Codakia rugifera* [11] *n* = 1, *Dulcina madagascarenis* [31] *n* = 3, *Epidulcina* cf*. delphinae* [31] *n* = 1, G*loverina vestifex* [31] n = 1, *L. aequizonata* [34] n = 7, *Lucinoma annulata* [34] *n* = 1, *Lucinoma atalantae* [24] *n* = 1, *Lucinoma borealis* [35] *n* = 6, *Lucinoma capensis* [36] *n* = 3, *Lucinoma kastoroae* [31] *n* = 2, *Lucinoma myriamae* [24] *n* = 3, *Monitilora ramsayi* [11] *n* = 2, *Myrtea* sp. [11] *n* = 1, *Myrtea spinifera* [37] *n* = 1, *Notomyrtea botanica* [11] *n* = 1, *Rostrilucina garuda* [31] *n* = 1, *Semelilucina semeliformis* [31] *n* = 1, *Taylorina solomonensis* [31] *n* = 1, *Troendleina suluensis* [22] *n* = 1, and *Ustalucina ferruginea* [38] *n* = 1. Habitat information such as depth were recorded when available and are found in supplementary Table S1.

Approximately 1 cm^2^ gill pieces were cut into 2 mm fragments and DNA was extracted using the Qiagen DNeasy Blood and Tissue kit (Cat. No. 69506; Qiagen, USA) or the animal tissue protocol from the Analytik Jena Innuprep DNA Mini Kit (Cat. No. 845-KS-1041250, Germany), following the manufacturers’ instructions with the following modifications: (1) Tissues were incubated in the lysis buffer solution provided in each kit until completely digested (2–20 h); (2) DNA was eluted in 50 µl of ultrapure water (Ref. 10977-015; Invitrogen, Life Technologies, USA). The extracted DNA was quantified using the Qubit dsDNA High Sensitivity Assay kit (Thermo Fisher Scientific, USA) and stored at −20 °C.

Sequencing library preparation, metagenomic and amplicon sequencing were carried out by the Joint Microbiome Facility of the Medical University of Vienna and the University of Vienna. Libraries were constructed using Illumina compatible library prep kits (NEBNext Ultra II FS DNA Library Prep Kit, NEBNext Ultra DNA v2 Library Prep Kit, Nextera Mate Pair Sample Preparation Kit). All libraries were sequenced with Illumina technology (HiSeq 3000, HiSeq 4000, and NovaSeq 6000) using paired-end settings with read-lengths of 100 bp, 150 bp, or 250 bp to generate a minimum of 1,000,000 reads (see Tables S1 and S2 for more details**)**.

Amplicon sequencing was performed on DNA from *B. thionipta* n = 5, *L. myriamae* *n* = 3, and *T. solomonensis* *n* = 1 to profile the bacterial communities in the gills of these species. The V4 regions of the 16S rRNA gene were amplified using the 515F and 806R primers [39, 40]. Library preparation, sequencing and downstream processing were carried out as described by Pjevac et al. [41]. ASV sequences were classified using DADA2 and the SILVA database SSU Ref NR 99 release 138.1 [42, 43] using a confidence threshold of 0.5. Samples that produced libraries of fewer than 1000 reads were sequenced a second time and only libraries larger than 500 reads were kept for the final analysis. Samples were visualized using the ampvis2 R package [44].

### Quality filtering, assembly, and bacterial genome binning

Read libraries were trimmed, PhiX contamination filtered, and quality checked using BBMap v37.61’s BBDUK [45] feature and the software’s adapter and PhiX databases to detect contaminants. All reads below an average q-score of 15 and a minimum length of 100 bp for 150 bp and 250 bp reads or 50 bp for 100 bp reads were removed. The minimum kmer size of 21 was used for trimming and reads matching the contaminants or below the quality score were filtered out. Libraries were interleaved as needed for future processing and analysis.

Individual read libraries were assembled using SPAdes v3.13.1 [46, 47] with the “metagenome” setting and iterative kmers of 21, 31, 41, 51, 61, 71, 81, and 91. All contigs shorter than 1000 bp were removed from the assembly prior to mapping and binning. Libraries from the same host and location (maximum of three, treated as biological replicates) were used for binning when possible. If three libraries from the same host and location were not available at the time of binning, binning was attempted with libraries from similar hosts or broad scale geographical areas. Read libraries were mapped to the scaffold assemblies using BBMap with default settings. The resulting sam files were converted to bam files with samtools v1.9 [48] and sorted using the anvi-init-bam script from Anvi’o [49]. The assembled scaffolds were then binned using a combination of Anvi’o v6.1 or 7.1 using CONCOCT v1.1.0 [50], with a minimum length of 1000 bp, and metaBAT v2.15 [51], with a minimum length of 1500 bp. A metaBAT binning attempt with no coverage information was also attempted outside of Anvi’o. All potential bins for each metagenome were then compared using dRep v2.4.2’s [52] dereplicate workflow with default settings and the “best” was selected automatically. If no bin was selected, a manual bin selection (using CheckM v1.1.3 [53] with Gammaproteobacteria gene set) and revision of the best Metagenome-Assembled Genome (MAG) using Anvi’o’s ‘anvi-refine’ was attempted to manually improve the quality of the MAGs. The bins were then checked for completion using CheckM’s taxonomy specific workflow using the previously mentioned gene set. MAGs that were more than 90% complete and 5% or less contaminated according to the gammaproteobacterial gene set in CheckM were used in further downstream analysis without further refinement. MAGs that failed the aforementioned quality thresholds (<90% complete and >5% contaminated) were manually refined using ‘anvi-refine’. MAGs that were 90% or more complete and less than 10% contaminated post-refinement, were deemed high-quality MAGs and kept for further analysis. Scripts for this workflow can be found in the supplementary material from Osvatic et al. [12].

### Phylogenetic analysis

All MAGs were taxonomically classified using GTDB v0.3.3 [54–56], and only the MAGs belonging to the *Sedimenticolaceae*, *Chromatiaceae*, and Thiohalomonadales were used for phylogenetic analyses. Publically available MAGs of previously described lucinid symbionts (*Ca*. Thiodiazotropha sp. and *Ca*. Sedimenticola sp.), alongside the MAGs of close relatives to *Ca*. Thiodiazotropha; *Ca*. Endoriftia persephone*, Sedimenticola selenatireducans, S. thiotaurini*, and *Allochromatium vinosum*, which was used as an outgroup for *Ca*. Thiodiazotropha MAGs, were downloaded from the NCBI database and included in the analysis (NCBI accession numbers in Table S4). MAGs related to Thiohalomonadales were also downloaded from NCBI (accession numbers contained within figures). All publicly available MAGs we used were quality checked using CheckM’s taxonomy specific workflow using the gammaproteobacterial set of marker genes.

All available MAGs, irrespective of CheckM’s estimated completion, were used to generate a concatenated multi-gene amino acid sequence alignment. *Ca*. Thiodiazotropha-associated genomes were run separately from Thiohalomonadales-related genomes to generate two phylogenies. The GTDB-Tk (Genome Taxonomy Database Toolkit, release 95) classify workflow was used to identify, align, and concatenate 120 highly conserved bacterial marker genes [57–62]. The concatenated amino acid sequence alignment generated from the GTDB-Tk workflow was submitted to the W-IQ-TREE server [63] using the default settings (auto substitution model detection, 1000 ultrafast bootstraps, and 1000 replicates of SH-aLRT branch tests). The final consensus tree of *Ca*. Thiodiazotropha was visualized and rooted with *A. vinosum* using Interactive Tree Of Life (iTOL) v5 [64]. The final consensus tree of the Thiohalomonadales phylogeny was visualized and rooted with Chromatiales bacterium GCA002007425 using Interactive Tree Of Life (iTOL) v5 [64]. The resulting phylogenetic trees were used alongside ANI analyses to identify species-level clades.

The average nucleotide identity (ANI) between MAGs was calculated using FastANI v1.3 [58] and gANI [65]. With FastANI, a program-suggested species cut-off of 95% one-way ANI was used. When FastANI values were near the 95% threshold, gANI was used as additional support. The gANI value was only used in select cases to confirm FastANI species groups. To do so, Prodigal v2.6.3 [59] was used to generate nucleotide sequences for all genes within individual MAGs, which were subsequently used within gANI to ANI values. The program-suggested cut-offs of 96.5% ANI and .6 alignment fraction in gANI were used for species delineation. These values were used to group MAGs into species groups and delineate closely related species. Species of interest were also assessed by the percentage of conserved proteins (POCP) method [66].

### MAG functional annotation

Open reading frames (ORF) in the symbiont MAGs were predicted using Prodigal v2.6.3 [59]. The protein-coding gene models were functionally annotated using eggNOG-mapper v2 with the eggNOG reference database v5.0 [67, 68]. All MAGs were also submitted to the RAST (Rapid Annotation using Subsystem Technology; https://rast.nmpdr.org/) web server for functional annotation using the default RASTtk pipeline [69]. Both sets of function annotations were manually curated for metabolic analysis and comparison. To search for missing genes of interest, ORFs were predicted for the initial raw metagenomic assemblies and the gene models were subsequently annotated using eggNOG. Word searches of the annotation tables were used to look for the presence of putatively missing genes. If a match was found, the target gene was then searched against the NCBI nr database using blastp [70]. If the best hits for the target genes shared the same taxonomic classification as the original MAG, this was used as evidence indicating that the target genes were present but have been excluded from the MAG during the binning process. As an additional layer of evidence to confirm the presence of missing target genes, orthologs of the missing target gene, obtained from the MAGs of a closely related species, were used as references for metagenomic read mapping using BBMap to calculate coverage rates (minimum identity threshold set to 90%).

### Estimating the strength of selection

To estimate the strength of selection, the non-synonymous to synonymous substitution rate ratio (dN/dS) in putative protein-coding genes was calculated for the high-quality MAGs of at least five individuals each for the following symbiont species: *Candidatus* T. endolucinida (*n* = 9), *Ca*. T. lotti (*n* = 10), *Ca*. T. weberae (*n* = 9), *Ca*. T. taylori (*n* = 10), *Ca*. T. “Aeq1” (*n* = 7), *Ca*. T. gloverae (*n* = 9), and the Thiohalomonadales symbionts of *B. thionipta* (Thiohalo2, *n* = 5). Substitution rates were calculated using ‘codeml’ in PAML v4.10.3 [71]. To ensure that our results could be accurately compared across different clades, a subset of 1000 putatively protein-coding genes (*i.e*., eggNOG annotations) were randomly selected to infer dN/dS ratios. Ratios of >1 indicate positive selection, whereas ratios around zero indicate neutrality. When considering values <1, lower values are a sign of strong purifying selection, while higher values are a sign of higher genetic drift and more relaxed selection [71]. The differences in dN/dS ratios between each symbiont species group were statistically tested using a non-paired, two-sample Wilcoxon Signed Rank Test in R v3.6.2. All scripts used for the selection analysis can be found on FigShare [72].

### Fluorescence in-situ hybridization

To visualize the gill endosymbionts of *L. myriamae* and *B. thionipta*, we carried out catalyzed reporter deposition fluorescence in-situ hybridization (CARD-FISH) using a Thiohalomonadales-specific probe with Cy3-labeled tyramides (Thiohalo845: 5′-TTAGCTTCGACACTAAGTCCT-3′).

Gill samples originally preserved and stored in >90% ethanol were embedded in paraffin wax by the Histopathology Facility at Vienna BioCenter Core Facilities, Austria. The paraffin-embedded tissues were cut into 5-µm sections with a Leica RM2235 Manual Rotary Microtome (Leica, Germany), and mounted on SuperfrostPlus adhesion slides (Thermo Fisher Scientific, USA). The sections were dewaxed in Roti-Histol (Carl Roth, Germany) three times for 10 min each, followed by two washes each in 99.9%, 96%, and 90% ethanol, and finally three washes in phosphate buffered saline (PBS) for five minutes before the slides were air dried.

The tissue sections were permeabilized in 0.2% HCl for 10 min and then washed PBS for 5 minutes and rinsed in sterile water. The sections were incubated in approximately 50 µl of lysozyme solution (10 mg/mL, TRIS-EDTA buffer) for 30 min at 28–30 °C, then washed in PBS and air dried for 5 min. Inactivation was performed by incubating slides in 0.1% H_2_O_2_ for 2 min, and followed by three washes in PBS. Hybridization was performed with 1 µl of 5 pmol/µl CARD-FISH probes (1×) in 299 µL hybridization buffer (HB, varying percentages of formamide; approximately 50 µl per section) for formamide series and binding assessments (Fig. S1). 30% Formamide was chosen as an optimal percentage. Specificity tests were performed using 30% formamide HB on a *L. myriamae* gill (Thiohalomonadales-containing, sample L130) and *Loripes orbiculatus* (*Ca*. Thiodiazotropha-containing, from Piran, Slovenia, not on sample list) to confirm that the probe did not bind to *Ca*. Thiodiazotropha rRNA (Fig. S2). Hybridization was finally performed with 1 µl of 5 pmol/µl CARD-FISH probes (1×) in 299 µL hybridization buffer (HB, 30% formamide; approximately 50 µl per section, Table S3). A nonsense-Eubacteria CARD-FISH probe was also used with each sample set to check for unspecific binding and autofluorescence (Fig. S1, Amann et al. [73]). The slides were incubated in humid chambers (50 ml tube containing paper wet with 1 mL of HB) at 46 °C for 3–4 h. The slides were subsequently washed twice in PBS followed by a 10 min incubation in PBS at 48 °C, before they were dried with compressed air. Signal amplification was performed using an amplification buffer (AB) containing 10 µL diluted H_2_O_2_ and 2 µL of a Cy3-labeled tyramide. Approximately 50 µL of AB was added to each section and incubated for 45 min in the dark at 46 °C. The slides were then washed twice in PBS and incubated in PBS for 10 min, followed by a brief wash in 50 mL 96% ethanol and air dried. Following CARD-FISH, the samples were DAPI-stained (1 µg/mL) and mounted in Citifluor antifade mounting media (Electron Microscopy Sciences, USA). All post-amplification steps were carried out in the dark. Images were captured on a Leica TCS SP8 X confocal laser scanning microscope using a 63× objective or a Leica DMi8 inverted microscope using a 20× objective.

## Results

### Deep-water lucinids host diverse symbiont lineages that are not found in shallow-water tropical clams

We sequenced, assembled, and binned metagenomes of 46 lucinid specimens representing 22 different host species from five subfamilies (24 Codakiinae, five Leucosphaerinae, nine Lucininae, two Monitilorinae, and six Myrteinae, Table S1). We retrieved 48 bacterial metagenome-assembled genomes (MAGs), 43 of which were deemed “high quality” because they were over 90% complete and had below 10% contamination (Table S4). Samples L181 (*B. thionipta*) and 3502H (*Epidulcina* cf. *delphinae*) were the only metagenomes that each yielded two high-quality MAGs (Table S4). All 48 MAGs were Gammaproteobacteria, 38 of which were assigned to the order Chromatiales and family *Sedimenticolaceae*, which contains all previously described lucinid symbiont species. The remaining ten MAGs were assigned to the order Thiohalomonadales and the SZUA-152 family, hereafter referred to as Thiohalomonadales (GTDB release 95, Table S4).

**Fig. 2:**
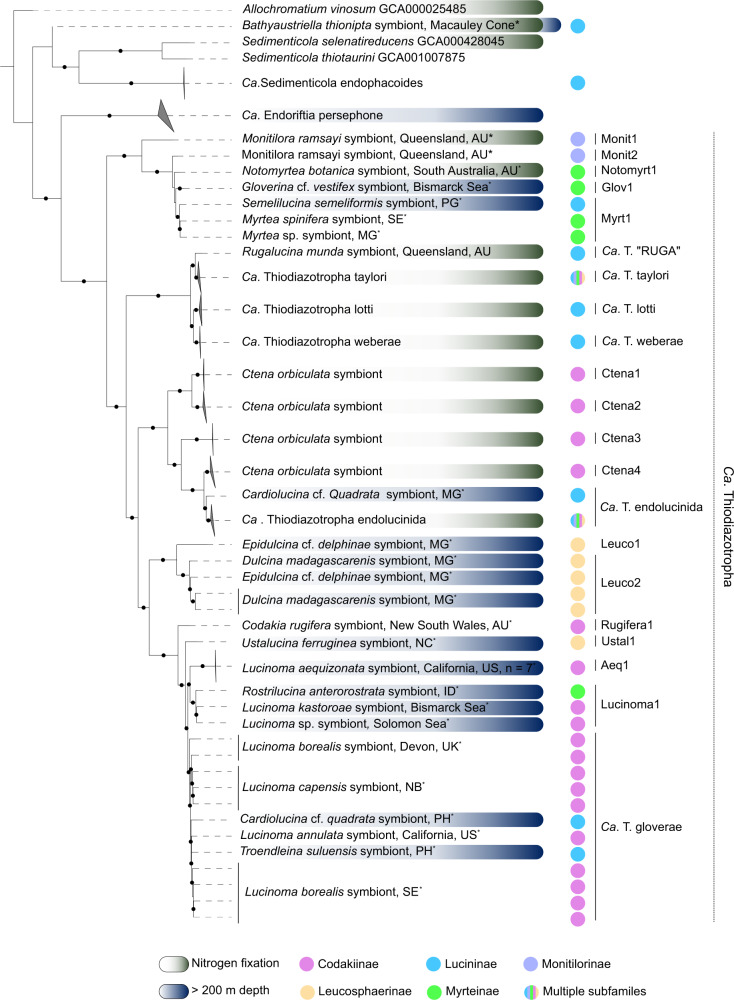
Deep-sea Lucinidae host divergent *Ca*. Thiodiazotropha. A maximum likelihood phylogenetic tree was reconstructed from GTDB’s multisequence alignment using the best-fit model LG + F + I + G4. Circles indicate bootstrap support values above 95%. Symbiont MAGs originating from the present study are indicated by the asterisk. Host subfamilies are labeled according to the color scheme used in the most recent molecular phylogenetic analysis of the Lucinidae [104]. Previously known symbiont species clades and novel species clades consisting of a single host and location were collapsed for ease of interpretation. Alignment and phylogeny are available on FigShare [105]. Geographical locations of *Ca*. T. taylori and *Ca*. T. gloverae samples are detailed in Fig. S3. Australia (AU), Papua New Guinea (PG), Madagascar (MG), New Caledonia (NC), Philippines (PH), United Kingdom (UK), US (United States of America), Sweden (SE), Namibia (NB).

**Fig. 3:**
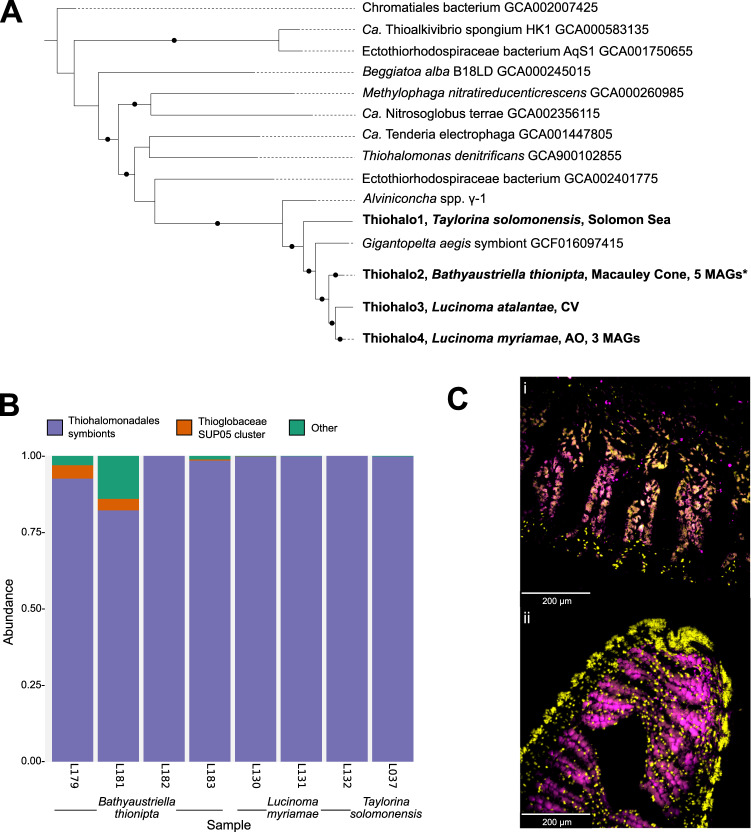
Several deep-water lucinid species host sulfur-oxidizing bacteria from the order Thiohalomondales that are closely related to the symbionts of hydrothermal vent gastropods. **A** Phylogenetic relationships of Thiohalomonadales assigned MAGs. A maximum likelihood phylogenetic tree was reconstructed from GTDB’s multisequence alignment of highly conserved bacterial marker genes using the best-fit model LG + F + I + G4. Circles indicate bootstrap support values above 95%. Novel species groups consisting of a single host and location were collapsed. The lucinid Thiohalomonadales symbionts are in bold font. Alignment and phylogeny are available on FigShare [106]. * MAGs encoding the nitrogen fixation pathway. **B** Relative abundance of ASVs within the gills of lucinids hosting Thiohalomonadales symbionts. **C** Distribution of bacteria within gills of (i) *Bathyaustriella thionipta* and (ii) *Lucinoma myriamae* detected using CARD-FISH. Probes specifically targeting Thiohalomonadales 16S rRNA (sequence in methods) - magenta, nuclei are stained using DAPI - yellow.

Phylogenomic analyses indicated that the symbiont MAGs represented 18 distinct bacterial species clades (13 *Ca. Thiodiazotropha*, one unclassified *Sedimenticolaceae* and four Thiohalomonadales), based on a 95% average nucleotide identity (ANI) threshold for species delimitation (Fig. 2 and Dataset S1–4). Twelve of the 13 *Ca. Thiodiazotropha* species clades comprised previously undescribed symbionts while a single MAG retrieved from a *Cardiolucina* cf*. quadrata* specimen (L029) was identified as *Ca*. T. endolucinida, a species previously known from shallow-water Caribbean lucinid hosts (Fig. 2, Tables S1 and S4, and Dataset S1). Nine of the 13 *Ca*. Thiodiazotropha species clades were symbionts harbored by lucinid species from deep-water habitats (>200 m, Fig. 2). Three symbiont species (“Myrt1”, “Lucinoma1”, and *Ca*. T. gloverae), all containing deep-water representatives, were associated with host species from multiple lucinid subfamilies (Fig. 2). *Ca*. T. gloverae, the most widespread and prevalent of the three species, was associated with *L. annulata, L. borealis*, and *L. capensis* (East Pacific, North and South Atlantic, respectively), and *Cardiolucina* cf. *quadrata* and *T. suluensis* from the Philippines Sea (Fig. 2). We proposed the name *Candidatus* Thiodiazotropha gloverae for this species after Emily Glover to honor her important contributions to understanding the biodiversity and evolution of the Lucinidae. Although one of MAGs retrieved from *B. thionipta* specimen L181 was assigned to the *Sedimenticolaceae*, this MAG was excluded from subsequent analyses because it was distantly related to the *Sedimenticola* and *Ca*. Thiodiazotropha genera, and present in only one of the five *B. thionipta* specimens (Fig. 2, Table S4, Supplementary Discussion). Finally, each of the four Thiohalomonadales symbiont species was associated with a distinct lucinid host species: *B. thionipta*, *T. solomonensis*, *L. atalantae*, and *L. myriamae*, from three lucinid subfamilies (Lucininae, Myrteinae, Codakiinae, and Codakiinae, respectively) (Fig. 3, Dataset S2–4). These Thiohalomonadales symbionts formed a clade with the “γ−1” symbiont of *Alviniconcha* sp. [2] and an unnamed symbiont (referred to as “Giga1”, GCF_016097415) of *Gigantopelta aegis* [74], both of which are hydrothermal vent gastropods (Fig. 3, Dataset S2–4). Analyses of the percentage of conserved proteins (POCP) and average amino acid identity, 42–50% and above 66%, respectively, both suggest this clade of bivalve and gastropod symbionts represented a single genus (Dataset S5) [66].

### Several deep-water lucinid species harbor sulfur-oxidizing bacteria from the order Thiohalomondales as intracellular symbionts

To better understand the bacterial communities associated with the four lucinid species from which Thiohalomonadales MAGs could be assembled (with one exception), we performed high-throughput sequencing of 16S rRNA amplicons with the eight individuals available. The results mirrored those from the metagenomes. The most dominant amplicon sequence variant (ASV) in each of these libraries was identical to the sequence found in the Thiohalomonadales MAGs from the corresponding specimen. In contrast to the metagenomic assignment to Thiohalomonadales based on GTDB, the 16S rRNA-gene amplicons were classified as “Thiomicrospiraceae endosymbionts” through the SILVA database. Considering GTDB’s more robust genomic database, we use this classification throughout. A maximum of two Thiohalomonadales ASVs were found per sample. The dominant ASV made up to 91.5% of the reads in all samples while the secondary Thiohalomonadales ASV comprised at most 8.1% (Fig. 3, Dataset S6). CARD-FISH with probes specific to the Thiohalomonadales bacteria on *L. myriamae* and *B. thionipta* gill sections, as well as previously published SEM micrographs of *B. thionipta* (additional SEM micrographs can also be found in Fig. S4), further indicate that bacterial endosymbionts are harbored internally within bacteriocytes in the gills (Fig. 3C) [25]. Finally, two ASVs were assigned to the SUP05 lineage of chemosynthetic sulfur-oxidizing bacteria typically associated with bathymodiolin mussels, but these were only present in low numbers: 4.4% and 3.7% of *B. thionipta* samples L179 and L181, respectively, and below 0.2% in samples L130 (*L. myriamae*) and L183 (*B. thionipta*) (Fig. 3, Dataset S6).

### Lucinid symbionts from diverse environments and phylogenetic lineages share the same core metabolic capabilities

Although there were minor differences within the pathways common to all the symbiont species, such as the presence/absence of different variants of the sulfide:quinone reductase (SQR) or ribulose 1,5-bisphosphate carboxylase/oxygenase (RuBisCO) proteins, symbionts from both the *Sedimenticolaceae* and Thiohalomonadales appeared to share the same core metabolic capabilities for oxidizing sulfur and fixing carbon that are fundamental to chemosynthetic symbiosis of the lucinid clams (Fig. S5). The symbionts’ ability to oxidize reduced sulfur compounds appears to be the basis for energy conservation in all so-far investigated lucinid symbioses. All *Sedimenticolaceae* and Thiohalomonadales symbiont MAGs encoded several pathways for sulfur oxidation, including at least two types of SQR and flavocytochrome-c-sulfide dehydrogenase (FCC) genes for oxidizing hydrogen sulfide to elemental sulfur, the truncated Sox system (*soxABYZ* genes), and the reverse dissimilatory-sulfate reduction (rDSR) pathway (Fig. S5, Tables S5, S6–7). The energy generated from sulfur oxidation is used to fix inorganic carbon through the Calvin–Benson–Bassham (CBB) cycle. All *Ca*. Thiodiazotropha symbionts MAGs encoded genes for either form I, II, or both of the RuBisCO enzymes (Tables S5–S8). In contrast, the Thiohalomonadales endosymbionts associated with lucinids, *Alviniconcha* spp. and *Gigantopelta aegis* possessed only RuBisCO form II, except the symbiont of *T. solomonensis* (“Thiohalo1”), which had an additional RuBisCO form I gene. In addition to these chemosynthesis pathways, all lucinid symbionts had the genomic potential for heterotrophic use of organic compounds, assimilating ammonia, storing phosphorous as polyphosphate, and respiring oxygen through cbb3-type cytochrome c oxidases (Fig. S5, Tables 1, S5–S7).

**Table 1:** Nitrogen processing metabolic potential of Lucinid symbiont species.

	*Sedimenticola*	*Ca*. Thiodiazotropha												Thiohalomonadales	
Feature	endophacoides	Myrt1	Taylori	Lotti	weberae	Ctena1	Ctena2	Ctena3	endolucinida	Leuco2	Aeq1	Lucinoma1	gloverae	Thiohalo2	Thiohalo4
Diazotrophy, nitrogenase			+	+	+	+	+	+	+*					+	
Respiratory nitrate reductase									+	+	+	+	+		
Periplasmic nitrate reductase	+	+	+	+	+	+	+	+	+	+		+	+	+	+
Assimilatory nitrate reductase			+	+	+	+	+	+	+					+	+
Nitrite reductase NADPH subunit		+	+	+	+	+	+	+	+	+		+	+	+	+
Copper-containing nitrite reductase (NO-forming)		+		+		+	+	+	+	+		+	+	+	+
Nitric oxide reductase	+	+	+	+	+	+		+	+	+			+	+	+
Nitrous-oxide reductase	+	+	+	+	+	+	+	+	+	+		+	+	+	+
Cyanate hydratase						+	+	+	+	+		+	+		
Urea metabolism	+		+		+										
Allophonate utilization			+	+	+										
Ammonia assimilation	+	+	+	+	+	+	+	+	+	+	+	+	+	+	+

Only lucinid symbiont species groups (*Sedimenticola*, *Ca*. Thiodiazotropha, and Thiohalomonadales) with three or more MAGs are shown. Smaller group information can be found in Tables S5 and S7.

+ gene(s) within a pathway are present in all the high-quality MAGs; -, gene(s) within the pathway were absent from all the high-quality MAGs.

* Genes are present in a majority of the MAGs but not all.

** Genes are not present in MAGs but predicted proteins from the unbinned metagenomes were found with the same function and a taxonomy within the same Order as the MAG.

### Nitrogen fixation capability is associated with shallow water

The ability to fix nitrogen through the *nif* genes is a hallmark trait of all previously described *Ca*. Thiodiazotropha symbionts associated with lucinids from seagrass and coral reef environments (Fig. 2, Tables 1, S6, and S7, Osvatic et al. [12]). Yet, none of the *Ca*. Thiodiazotropha symbionts of clams from locations deeper than 50 m had the core functional genes for nitrogen fixation (Table 1, Fig. 2, Tables S6–8). Furthermore, although all shallow-water representatives of *Ca*. T. endolucinida possessed the entire complement of *nif* genes [12], the *Ca*. T. endolucinida MAG associated with the *Cardiolucina* cf. *quadrata* specimen (sample L029), from deep-sea sediments (230–288 m) off Madagascar, only possessed *nifJ* and *nifZ* (Table S1). Metagenomic reads from this sample did not map to any of the other *Ca*. T endolucinida *nifABEHMNQWZ* genes (GCA001715975), further suggesting that this deep-water *Ca*. T. endolucinida strain has lost the ability to fix nitrogen (Tables S6, S9, and S10). In particular, the gene annotated as *nifJ*, in particular, may not only be involved in nitrogen fixation as KEGG pathway reconstruction classified this gene as a pyruvate-ferredoxin/flavodoxin oxidoreductase (E.C 1.2.7.1) involved in central carbon metabolism and did not assign this gene to the nitrogen fixation pathway. These findings indicate that multiple lineages in the *Ca*. Thiodiazotropha genus lack the ability to fix nitrogen and the presence or absence of this ability is correlated with environmental factors, more specifically, its absence is associated with living in a deep-sea habitat. Similarly, nearly all members of the symbiotic Thiohalomonadales clade never possessed more than two components of the *nif* operon, and if they did it was primarily *nifJ*, indicating that the endosymbionts of deep-water lucinids and hydrothermal vent gastropods were unable to fix nitrogen. The sole exception was the “Thiohalo2” species clade associated with *B. thionipta*, which had a complete *nif* operon apart from *nifF* (Tables 1, S6, and S7). In particular, the “Thiohalo2” *nifBDEKM* genes, in particular, shared high amino acid sequence identity with their *Ca*. Thiodiazotropha orthologs. Although the “Thiohalo2” *nifH* shared highest identity with an unannotated gene from a member of the Zetaproteobacteria (92%), it nevertheless shared 90.7% pairwise amino acid sequence identity with *Ca*. Thiodiazotropha *nifH* genes (Dataset S7 and Fig. S7).

All previously described lucinid symbionts have the potential for utilizing alternative nitrogen sources. Apart from the symbionts of *L. aequizonata* (“Aeq1”), all Thiohalomondales and *Ca*. Thiodiazotropha symbionts possessed a periplasmic nitrate reductase for dissimilatory nitrate reduction and a nitrite reductase NADPH subunit for assimilatory nitrate reduction (Tables 1, S6, and S7). Most symbiont MAGs also encoded the complete denitrification pathway for reducing nitrate to N_2_, with the exceptions of *Ca*. T. weberae and *Ca*. T. taylori, which lacked copper-containing nitrite reductase, and “Ctena2”, which was lacking nitric oxide reductase (Tables 1, S6, and S7). The “Aeq1” symbiont was particularly noteworthy in being the only symbiont to have lost all denitrification genes except the respiratory nitrate reductase (Tables 1, S6, and S7). Osvatic et al. [12] previously reported that *Ca*. T. taylori, *Ca*. T. weberae, *Ca*. T. “RUGA”, *Ca*. T. lotti had the potential for using urea, but genes for the urease and allophanate pathways were absent from the MAGs of all the symbionts described here (Tables 1, S6, and S7). Several symbiont species did however have genes for exploiting other nitrogen sources such as cyanate; the MAGs of *Ca*. T. endolucinida, *Ca*. T. gloverae, “CTENA1-4”, “Ustal1”, “Leuco2”, and “Lucinoma1”, from shallow and deep-water environments, encoded a gene for the cyanate hydratase enzyme, which converts cyanate into carbon dioxide and ammonia.

The methylotrophy pathway, which enables growth on single carbon compounds, (genes *pqqBCDE*, *mxaF*, *exaA*, *exaB*, and the one-carbon transfer pathway utilizing methanofuran) was previously found in multiple shallow-water *Ca*. Thiodiazotropha species (Tables S6 and S7) [12]. Most of these genes were present in the deep-water *Ca*. T. endolucinida MAG (*Cardiolucina* cf*. quadrata* symbiont) and in the majority of *“*Lucinoma1” and *Ca*. T. gloverae MAGs, as well as in “Rugifera1”, a *C. rugifera* symbiont closely related to “Lucinoma1” (Tables 1, S5–S7). In contrast, none of the Thiohalomonadales symbionts in this study or described previously (*Alviniconcha* spp. and *Gigantopelta aegis* symbionts) had the capacity for methylotrophy (Tables S5 and S7).

### The symbionts of lucinids from extreme environments are under strong purifying selection

**Fig. 4:**
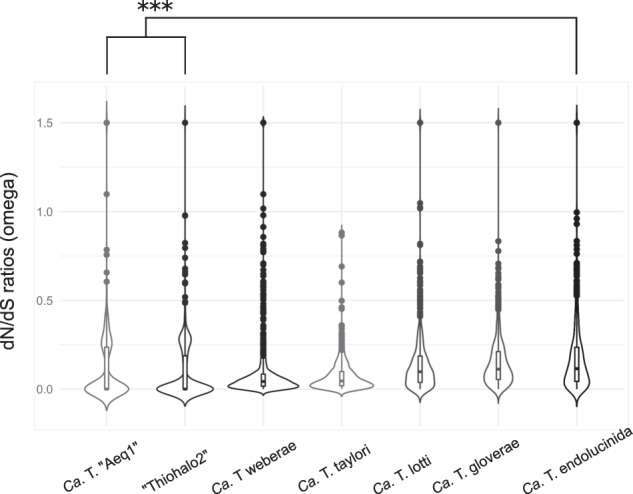
The genomes of the *L. aequizonata* and *B. thionipta* symbionts experienced the strongest purifying selection. Adaptive evolution in protein-coding sequences of lucinid symbiont genomes was inferred using the ratio of non-synonymous to synonymous substitutions (dN/dS ratio; omega). Only symbiont species groups with five or more high-quality MAGs were used for this analysis. The distribution of dN/dS ratios is shown with a violin plot surrounding a boxplot of the median omega value of each species. The omega values of *Ca*. T. “Aeq1” and “Thiohalo2” were significantly lower than all other species analyzed (***p < 0.001, two-sample Wilcoxon Signed Rank Test corrected for multiple testing).

The median dN/dS ratio (omega) values for the symbionts of *L. aequizonata* and *B. thionipta* (*Ca*. T. “Aeq1” and Thiohalo2, respectively) were the lowest of all the symbiont species analyzed (both 0.002, Two-sample Wilcoxon Signed Rank Test, *p* < 0.001, Fig. 4, Table S11). The median omega values of *Ca*. T. taylori and *Ca*. T. weberae were 0.048 to 0.072, respectively, while the median omega values of *Ca*. T. endolucinida, *Ca*. T. gloverae, and *Ca*. T. lotti were the highest and ranged from 0.091 to 0.123 (Fig. 4, Table S11). There was no significant difference in the media omega values of the latter three symbiont species, but they were significantly higher than all other symbiont species tested (Table S11). There was also no significant difference in the median omega values between *Ca*. T. taylori and *Ca*. T. weberae. Therefore, *Ca*. T. “Aeq1” and Thiohalo2, two symbiont species originating from oxygen-minimum zone and hydrothermal vent habitats, respectively, experienced the highest levels of purifying selection of all the symbiont clades analyzed (Fig. 4, Table S11). We did not observe any trends indicating a correlation between habitat depth and strength of selection. For example, *Ca*. T. weberae and *Ca*. T. taylori, both of which inhabit shallow-water environments, had significantly different omega values, while *Ca*. T. gloverae and *Ca*. T. lotti had similar dN/dS ratios despite their very distinct geographic and depth distribution patterns (Fig. 4, Table S11). Finally, the median omega values of endemic symbionts (*Ca*. T. weberae, *Ca*. T. lotti; 0.067) were not significantly different from cosmopolitan symbionts (*Ca*. T. gloverae, *Ca*. T. taylori; 0.075, *p* > 0.05, W = 523,558).

## Discussion

### Lucinids from vastly different environments associate with symbionts from the genus *Ca*. Thiodiazotropha

Lucinid clams have radiated across diverse habitats in Earth’s oceans, from the intertidal zone to hydrothermal vents in the deep sea. So far, our understanding of the lucinid symbiosis stemmed largely from a limited variety of shallow-water species, thus, little was known about the role their characteristic symbiosis played in colonizing harsh, deep-sea environments. Here, we sequenced the gill metagenomes of 22 lucinid species from a myriad of primarily deep-water habitats across the globe (Fig. 1 and Table S1), and retrieved 48 symbiont MAGs, from which we identified twelve undescribed symbiont species from the genus *Ca*. Thiodiazotropa that have not been previously found in lucinids from shallow tropical environments. Some of these species (*Ca*. T. gloverae, *Ca*. T. endolucinida, and “Myrt1”) were globally distributed and had extensive water depth ranges, while others appeared limited to specific geographic locations or environments (Figs. 1, 2, Tables S1 and S4). These findings highlight the unappreciated diversity of the *Ca*. Thiodiazotropha genus, their widespread presence in diverse marine environments with distinct geochemical conditions, and further show that lucinids from diverse phylogenetic lineages, living in distinct habitats and conditions, all prefer associating with symbionts stemming from a single, closely related group of sulfur-oxidizing bacteria from the family *Sedimenticolaceae*. Even *M. ramsayi*, a member of the earliest-branching lucinid lineage (Monitilorinae) sampled in our study, was associated with *Ca*. Thiodiazotropha symbionts. This conservation of the lucinid-*Ca*. Thiodiazotropha association across five lucinid subfamilies of varying evolutionary age, suggests that this ancient association dates back to the last common ancestor of the Monitilorinae and other lucinids. However, there was no clear evidence of congruence between host evolutionary history and symbiont phylogeny. Diverse *Ca*. Thiodiazotropha species, particularly those with global distributions like *Ca*. T. gloverae, frequently associated with multiple divergent host lineages, which is consistent with a strategy of acquiring symbionts horizontally from the environment (Fig. 2). These findings indicate that lucinids from different habitats have access to a diverse and widely distributed pool of candidate symbionts from the *Ca*. Thiodiazotropha genus, and further highlights the immense capacity of these bacteria to power symbiotic chemosynthetic primary productivity under vastly different environmental conditions. Moreover, the flexibility in the lucinid symbiont pairing increases the likelihood of forming a successful association across diverse environments and is likely to be an important contributing factor underlying the evolutionary success of this chemosynthetic symbiosis.

### Environmental factors such as temperature, nitrogen, and oxygen availability shape symbiont distribution and metabolic functions

Although the genetic diversity of environmental symbiont populations has never been thoroughly investigated, presumably, the environment has a significant impact on symbiont distribution and genomic potential, influencing which symbiont types are available to the lucinids in a particular habitat. Hence, it has been suggested that hosts acquire symbiont genotypes adapted to the local environment [75]. The selection of locally adapted symbionts could explain why half of the 12 *Ca*. Thiodiazotropha species described here appear to have limited geographic ranges (Fig. 2), but these distribution patterns could also be biased by limitations in sampling effort. Furthermore, the recent discovery of symbiont species associated with lucinids from distant geographic locations around the world challenges this paradigm [12]. *Ca*. T. gloverae, for example, was associated with lucinid species from across the Philippines Sea, Pacific Ocean, North Sea, and Atlantic Ocean (Fig. 2, Tables S1 and S4). Though this cosmopolitan distribution is not unprecedented (see [12]), *Ca*. T. gloverae also occurred in a wide range of habitats, from sediments at 740 m depth close to the equator, to intertidal seagrass meadows in Northern Europe (Fig. 2, Tables S1 and S4). It is therefore intriguing that *Ca*. T. gloverae does not co-occur with *Ca*. T. taylori, another globally distributed lucinid symbiont that only associates with lucinids from tropical shallow-water seagrass and reef environments [12]. Although, we are unable to determine the precise factors driving these distribution patterns without direct empirical measurements of local environmental conditions, it is interesting to speculate that the non-overlapping ranges of the globally distributed *Ca*. T. gloverae and *Ca*. T. taylori could be driven by their adaptation to distinct temperature regimes that vary with depth and latitude. Future studies should thus take into temperature as a potential factor shaping the environmental pool of *Ca*. Thiodiazotropha genotypes available as potential candidates for the lucinid symbiosis.

We also observed that nearly all *Ca*. Thiodiazotropha symbionts of clams from cooler temperate or deeper waters around the world were missing the *nif* gene cluster encoding the nitrogen fixation pathway, a metabolic feature that is ubiquitous in their shallow-water tropical relatives. This corroborates the previous unsuccessful attempts to amplify the *nifH* gene in samples of *Epidulcina* cf*. delphinae* and *L. borealis* [13, 76]. Even the deep-water (250 m) *Ca*. T. endolucinida strain associated with *Cardiolucina* cf*. quadrata* (Madagascar) lacked a *nifH* or any of the functionally important nif genes that were detected in genomes from the same symbiont species from the Caribbean [12, 19] (Fig. 2 and Tables S5–7). However, all lucinid symbionts described thus far have the ability to assimilate ammonium and some also possessed an assimilatory nitrate reductase, which are more economical strategies to acquire nitrogen than energetically expensive nitrogen fixation [77–81]. It is also worth mentioning that although periplasmic nitrate reductase is not typically associated with nitrate assimilation, Sanders et al. previously proposed an alternative mechanism by which ammonia produced through periplasmic nitrate reduction and subsequent cytoplasmic nitrite reduction could potentially be exploited for biosynthesis [82]. There are no measurements of inorganic nitrogen concentrations directly in the environments sampled for this study, but nitrate levels can reach 40 μM and ammonium can reach millimolar concentrations in deep ocean waters [77–81], while these are typically below 10 μM in tropical seagrass beds and 180 μM in coral reefs, both oligotrophic and nitrogen-limited [19, 83–85]. Furthermore, many of the lucinids in the present study occurred in continental shelf or temperate coastal environments that typically receive significant seasonal influxes of organic matter from either upwelling processes or terrestrial runoff, which can result in elevated levels of nitrate and ammonium in the water column and sediments [86, 87]. Hence the absence of nitrogen fixation in the symbionts of lucinids from temperate shallow-water and deep-water environments suggest either a sufficient supply of inorganic nitrogen in the environments or that these hosts to rely more heavily on heterotrophic filter-feeding to meet the metabolic nitrogen requirements of the symbiosis. The reliance on heterotrophic filter-feeding versus inorganic nitrogen is likely to depend on local conditions. For example, *Lucinoma* from oxygen-minimum zones (OMZs) are likely to inhabit organic matter-rich sediments that are low in ammonium [88–90], while the mid-oceanic ridge hydrothermal vent environments inhabited by *B. thionipta* are typically replete with nitrate but limited in ammonium and organic matter [82, 91]. These lines of evidence lead us to conclude that the nitrogen budget of the ecosystem is an important factor influencing the metabolic functions of the lucinid symbiosis.

Meeting the oxygen demand of their symbionts’ chemoautotrophic metabolism can be a flux-limiting physiological challenge under hypoxic or anoxic conditions (reviewed by Childress and Girguis, 2011 [92]). However, two of the lucinid species in the present study, *L. aequizonata* and *L*. capensis, have evolved to live in the low oxygen conditions in the oxygen-minimum zone (OMZ) sediments off the Californian and Namibian coasts. *L. aequizonata* and *L. capensis* are associated with two distinct but closely related symbiont species, the “Aeq1” symbiont clade and the globally widespread *Ca*. T. gloverae, respectively (ANI > 91%; Dataset S1). Despite their close phylogenetic relationship, the “Aeq1” MAGs were characterized by several highly derived traits: (1) the “Aeq1” MAGs have undergone genome streamlining and are substantially smaller (ranging from 2.9–3.2 megabases) than all other hiqh-quality *Ca*. Thiodiazotropha MAGs (average 4.63 megabases) (Table S4); (2) Consistent with their reduced genome size, “Aeq1” has lost key biological and metabolic capabilities, such as flagellar biogenesis, and enzymes involved in multiple nitrogen metabolic processes, including assimilatory nitrate and nitrite reduction and denitrification (Nir, Nor, Nos), all of which are present in the *Ca*. T. gloverae MAGs (Fig. S7, Tables S5 and S7). However, conservation of the respiratory nitrate reductase gene indicates that the “Aeq1” symbionts can use nitrate as a terminal electron acceptor instead of oxygen. These genomic data are consistent with previous physiological experiments indicating that the *L. aequizonata* symbionts reduce nitrate to nitrite but no further [8, 93, 94]. Interestingly, *Ca*. T. gloverae, the *L. capensis* symbiont, also has the ability to respire nitrate and it was recently shown that *L. capensis* has a three-fold higher nitrate consumption rate compared to other mollusc species that co-occur in the Namibian OMZ [95] (Table 1). Partnership with symbionts that respire nitrate could provide the lucinid symbiosis an important evolutionary advantage in an OMZ environment, as it prevents competition between the symbiotic partners for the scant amounts of oxygen that might be available.

### Rare cases of symbiont switching to the Thiohalomonadales correspond to major habitat changes

Nearly all members of the Lucinidae, including the species from the diverse habitats and phylogenetic lineages investigated in the present study, have specifically established symbiosis with selected symbionts from only a single group of bacteria – the family *Sedimenticolaceae*. We nevertheless discovered four deep-sea (315–2130 m) lucinids from divergent subfamilies and distant geographic locations that were exceptions to this rule: *T. solomonensis* (Myrteinae), *B. thionipta* (Lucininae)*, L. atalantae*, and *L. myriamae* (Codakiinae), which have all independently acquired closely related symbionts from the order Thiohalomonadales (Fig. 3 and Table S4). Both 16S rRNAgene and phylogenomic analyses indicate these Thiohalomonadales symbionts are closely related to the sulfur-oxidizing (SOX) symbionts of diverse animals that inhabit hydrothermal vents environments, including the bivalve *Maorithyas hadalis* (Thyasiridae) and the gastropods *Alviniconcha* spp. (Provannidae; “γ−1” symbiont), and *Gigantopelta aegis* (Peltospiridae) [2, 74, 96]. Only one of the four lucinids associated with Thiohalomonadales symbionts, *B. thionipta*, was from hydrothermal vent sediments [25], while *L. myriamae* inhabits a cold-seep environment and we have no habitat data for the other two species [24]. The paucity of environmental data stemming from these deep-sea lucinid habitats makes it impractical to determine the drivers underlying these symbiont switching events, but lab experiments on hydrothermal vent snails associated with the “γ−1” symbiont, which is closely related to the lucinid Thiohalomonadales symbionts, could provide us some clues. In the Lau basin, the *Alviniconcha* snails associate with either Campylobacteria (*A. boucheti*) or Gammaproteobacteria (“γ−1” symbiont of *A. kojimai, A. strummeri*) SOX bacteria, and it was found that the “γ−1” symbiont was better than the Campylobacteria symbiont at fixing carbon under low H_2_S and H_2_ concentrations, which suggests the “γ−1” symbiont is physiologically adapted to hydrogen- and sulfide-limited niches [94]. We are currently unable to determine whether these lucinids have selected Thiohalomonadales symbionts because they are better adapted to low reductant concentrations in these deep-sea habitats or if it is because members of the *Ca*. Thiodiazotropha and associated *Sedimenticolaceae* were in abundances too low for lucinids to establish or maintain a viable gill symbiotic community. Although the selective drivers are unclear, it is likely that these symbiont switches have coincided with major shifts to new habitats with environmental conditions that are not typically experienced by other members of the Lucinidae. Such habitats might be characterized by more dynamic biogeochemical conditions, and the formation of steep geochemical and temperature gradients. Our findings thus highlight a need for more extensive research encompassing the diversity of lucinids across the full spectrum of their natural habitats coupled with physiological experiments and environmental measurements to determine whether acquiring a locally adapted symbiont indeed provides the lucinid host with a fitness advantage.

The genomes of symbiont species from two “extreme” habitats, “Thiohalo2” and *Ca*. T. “Aeq1” associated with the hydrothermal vent dwelling *B. thionipta* and oxygen-minimum zone dwelling *L. aequizonata*, respectively (Figs. 1, 4), exhibited the highest levels of purifying selection, which also correlated with relatively small genome sizes: *Ca*. T. “Aeq1” had a mean genome size of 3 megabases, compared to an average of 4.63 megabases across the other *Ca*. Thiodiazotropha species, while “Thiohalo2” had a mean genome size of 3.56 megabases (Table S4). “Thiohalo2”, however, did not have a smaller genome size than the other Thiohalomonadales. We did not observe any relationship between habitat depth and degree of selection pressure (Fig. 4). We posit that genome streamlining due to selection for more efficient use of nutrients in populations with a large effective size could explain these patterns, rather than genome reduction due to genetic drift associated with small effective population sizes, as is typical of insect endosymbionts [97]. Whether selection acts on the free-living or symbiosis stage cannot be concluded with the data available, but given that no release of symbionts from the host to the environment has ever been observed [98], selective pressure during the free-living life stage seems more likely. We speculate that symbiosis with a lucinid host in harsh environmental conditions could provide a means of escaping nutrient limitation during the free-living stage.

### Why is symbiont switching away from the genus *Ca*. Thiodiazotropha so uncommon?

The flexibility to partner up with completely different groups of sulfur-oxidizing bacteria like the Thiohalomonadales, is clearly an important strategy that allows lucinids to exploit novel niches and could be critical to survival in more extreme environments like hydrothermal vents. An additional noteworthy example of symbiont switching is the association between *Ca*. Sedimenticola endophacoides, the only lucinid symbiont known in this genus, and its host *Phacoides pectinatus*, which lives deeply burrowed in sulfide-rich intertidal mangrove mud [15] (Table 1), one of the more extreme and oxygen-limited shallow-water lucinid habitats. Symbiont shifts in terrestrial plants and invertebrates are typically associated with trophic shifts [99, 100], while in the marine environment, variations in symbiont carbon and nitrogen metabolic potential similarly appear to shape the nutritional symbioses between siboglinids and chemosynthetic bacteria to allow the colonization of and adaptation to different chemosynthetic environments [101]. Although carbon and nitrogen metabolisms are variable across and even within the Thiohalomondales, *Ca*. Thiodiazotropha and *Ca*. Sedmenticola, we have not observed strong correlations between the variability in these metabolic capabilities and host habitat or geography (Table 1). Furthermore, the discovery of multiple globally distributed symbiont species shared by lucinids from diverse environments inevitably raises the question of whether lucinids derive any benefit from associating with a locally adapted endemic symbiont genotype instead of a cosmopolitan species. For example, even though nitrogen fixation is a nearly ubiquitous feature in shallow-water *Ca*. Thiodiazotropha, it remains unclear whether the lucinids associate almost exclusively with diazotrophs because these symbionts confer a fitness advantage in these environments or if this trait is simply ubiquitous in the pool of potential candidate symbionts living in an oligotrophic habitat, where selective pressures are exerted by nitrogen limitation. Indeed, a multitude of lucinid species clearly thrive without associating with a diazotrophic symbiont, and furthermore, the ability to filter-feed (a strategy used by lucinids and other animals with autotrophic symbionts) could potentially provide sufficient nitrogen for growth in oligotrophic environments. The reduced metabolic repertoire of the “Aeq1” clade further illustrates that relatively few fundamental metabolic pathways, most of which involve sulfur oxidation and carbon fixation, are in principle required for the functioning of the lucinid symbiosis. It is notable that in most environments, lucinid fitness does not depend on the specialized metabolic features of a locally adapted and/or endemic symbiont genotype. Non-dietary ecological factors such as thermal sensitivity can also shape symbioses, and the replacement of thermally intolerant symbionts with tolerant strains has been shown in aphids exposed to elevated temperatures in laboratory settings [102]. Instead, we suggest that future work should investigate whether acquiring symbionts that can maintain optimal chemosynthetic productivity under the temperature regime of the local environment affects the functioning and success of the lucinid symbiosis.

Although symbiont switches have occurred multiple times in the lucinid family, losses of *Ca*. Thiodiazotropha symbionts are nonetheless rather uncommon. While lucinid hosts appear to freely associate with diverse *Ca*. Thiodiazotropha genotypes, it is possible that physiological incompatibilities constrain the formation of partnerships with more phylogenetically distant sulfur-oxidizing bacteria. A lack of shared evolutionary history can result in decreased fitness as has been demonstrated in *Steinernema* nematodes following switches to a closely related but non-cognate symbiont [10]. These initial incompatibilities could potentially be ameliorated through mechanisms like carrying a greater symbiont load to compensate for the new symbiont’s weaker performance [103]. Alternatively, our findings suggest there is likely a rich genetic pool of candidate symbionts in the environment with an extensive metabolic repertoire. These diverse metabolic capabilities likely allow the *Ca*. Thiodiazotropha genus to persist across a myriad of environments characterized by distinct geochemical conditions, which could be an important factor contributing to their global success and explain why symbiont switching events are a relatively rare occurrence among the lucinids.

## Supplementary information

Supplemental Material

Supplementary Tables

Supplementary Dataset_S1

Supplementary Dataset_S2

Supplementary Dataset_S3

Supplementary Dataset_S4

Supplementary Dataset_S5

Supplementary Dataset_S6

Supplementary Dataset_S7

## Data Availability

The raw reads and MAGs have been deposited in the NCBI BioProject database (https://www.ncbi.nlm.nih.gov/bioproject/) with links to BioProject accession numbers PRJNA744034 and PRJNA765502. The BioSample accession numbers for the MAGs are SAMN20122384-SAMN20122425 and SAMN21561986-SAMN21561989, while SAMN20119405-SAMN20119448 and SAMN21572993-SAMN21572996 belong to their corresponding raw read sets. Amplicon sequences are also found under PRJNA744034. Phylogenetic trees and corresponding alignments are available on FigShare with information accompanying corresponding figures. All other study data are included in the article and/or supporting information files.
