# Supplementary material for: Gene loss and symbiont switching during adaptation to the deep sea in a globally distributed symbiosis
Source: ISME J. 2023 Jan 13;17(3):453–66. doi: 10.1038/s41396-022-01355-z (PMC9938160; doi:10.1038/s41396-022-01355-z)
Supplement: Supplementary file 1 — Supplemental Material [file 41396_2022_1355_MOESM1_ESM.pdf]

Supplemental Information

SI Methods

Nif gene blast searches and tree generation

To determine phylogenetic relationships of nif genes from Thiohalomonadales, Nif-operon genes from Thiohalomonadales MAGs found within *Bathyaustriella thionipta* (sample IDs L179-L183) were taken from RAST and aligned to determine sequencing identity to each other. Protein sequences from a single MAG (sample ID L183) were then blasted against all available sequences on NCBI using BlastP[1], with default settings. The top 50 hits from BlastP were used to generate a phylogeny using MAFFTv7.427[2] to generate an alignment, FastTree v2.1.11[3] to create the phylogeny, and were visualized on the iTOL web server[4].

Scanning electron microscopy

Lucinoma borealis (from Devon, UK, Table S1) and *Rugalucina munda* (from Tin Can Bay, Australia, Table S1) gill segments were fixed in 2.5% glutaraldehyde in PBS. *Bathyaustriella thionipta* and *Lucinoma myriamae* were preserved in 90% ethanol. Gill segments of *L. borealis* (Table S1) and *R. munda*, pieces of gill were sliced with a razor blade, critical point dried, mounted on stubs and sputter-coated with gold before scanning electron microscopy and visualized. *B. thioniptia* (reproduced from Glover et al., 2004[5]) and *L. myriamae* gills were dissected, pieces of gill were sliced with a razor blade, mounted on stubs and sputter-coated with gold before scanning electron microscopy and visualized.

SI Results

Lucinoma aequizonata symbionts lack metabolic capabilities encoded by most other *Ca*. Thiodiazotropha symbionts

Seven high-quality *Ca.* Thiodiazotropha MAGs (from seven different host specimens) were recovered from *Lucinoma aequizonata*. These MAGs, assigned to species group Aeq1, averaged 3.03 megabases, ranging from 2.86-3.18 megabases, making it the species group with the second smallest MAG/genome. Aeq1 species MAGs were the only species found within *Lucinoma aequizonata* metagenome samples; no secondary or co-occuring symbionts were detected. The average Aeq1 MAG/genome size was only second to a single *Ca.* Thiodiazotropha MAG from *Gloverina* cf. *vestifex*, assigned to the Glov1 species group (2.56 megabases). The average of 3.03 megabases is 1.60 megabases smaller than the average (4.63 megabases) hiqh-quality *Ca.* Thiodiazotropha MAG analyzed in this study.

Aeq1 MAGs, like all other MAGs categorized as *Ca.* Thiodiazotropha, encoded for components considered vital to the lucinid symbiosis. These components include a RuBisCo gene, in this case form II, as well as genes for hydrogen sulfide and sulfur metabolism (Table S5). These basic components are found in all Aeq1 MAGs. Aeq1 MAGs have a noted lack of metabolic capabilities found in close relatives (Lucinoma1 and *Ca.* T. gloverae). Lucinoma1 and *Ca.* T. gloverae encoded for complete denitrification pathways, while Aeq1 MAGs only encoded for a respiratory nitrate reductase, indicating that Aeq1 species can only utilize nitrate and not nitrite, nitric oxide, nitric oxide, or atmospheric nitrogen. However, Aeq1 bacteria can generate a proton-motive force for energy through the respiratory nitrate reductase, which is lacking in Lucinoma1 and *Ca.* T. gloverae (Table S5 and S6). Aeq1 MAGs also do not encode for main components of the flagella and no fli or flg genes were found in the MAGs. This represents a

major distinction between Aeq1 MAGs and all other *Ca.* Thiodiazotropha MAGs, as all others encoded for a majority of the flagellar-associated genes that were investigated.

Thiohalomonadales nitrogen gene phylogeny

Complete nitrogen fixation pathways were found within all Thiohalo2 species MAGs from the host species *Bathyaustriella thionipta*. All searched nif genes (nifBDEHKM) in these MAGs were 100% identical on a nucleotide level and therefore, genes from only one representative MAG were searched on NCBI's BlastP. A majority of the top 50 hits from each gene were identified as Gammaproteobacteria (Dataset S7 and Figure S6). All genes, except nifH, were closely related to genes from members of the Sedimenticolaceae family, consistent with 16S rRNA and genome phylogenies. In contrast, the nifH gene was most closely related to sequences from Zetaproteobacteria, possibly indicating acquisition of the *nifH* gene by horizontal gene transfer in these symbionts.

SI Discussion

Secondary Sedimenticolaceae MAG in *Bathyaustriella thionipta* distantly related to other known lucinid symbionts

A single Bathyaustriella thionpta specimen (L181) yielded two MAGs, one classified as belonging to the family Sedimenticolaceae, which contains all known lucinid symbionts from previous studies, and one classified as belonging to the order Thiohalomonadales, the novel symbiont group discovered here for the first time. Both were defined as high-quality MAGs (> 90% completion and <5% contamination, Table S4). This raises the question of whether the Thiohalomonadales MAG is simply a 'contaminant' of these samples, and the Sedimenticolaceae MAG represents the true endosymbiont. However, several observations

point to the Thiohalomonadales MAG being the dominant endosymbiont in this host species. These observations included included a) MAGs from same Thiohalomonadales genus were found in metagenomes from multiple lucinid species and has been shown to be an endosymbiont in other hydrothermal vent organisms, b) it was consistently found in all *B. thionipta* metagenomes, in contrast to the Sedimenticolaceae MAG, which was only sporadically found, and c) A Thiohalomonadales ASV identical to the MAGs from this genus dominated 16S rRNA gene sequencing libraries from all samples of this host species.

One possible explanation for the presence of a Sedimenticolaceae MAG in a metagenome from one *B. thionipta* individual could be that it was due to cross-contamination during multiplexed metagenome sequencing. We consider this unlikely, as it is highly distinct from all other MAGs sequenced during this project. Moreover, an ASV that perfectly matched this MAG made up 12.8% of the amplicon reads from this individual. It is therefore likely that this is a ‘real’ organism that was contained in the sample from *B. thionipta*. It is common for co-occurring symbiont to be found to some degree in ASV surveys of lucinid gills, even if they are missing from corresponding metagenomes (e.g. Lim et al., 2019[6]; Osvatic et al., 2021[7]). Still, its affiliation with the Sedimenticolaceae family, along with other known symbionts of lucinid clams, raises the possibility that this is a novel species of chemoautotrophic gill symbionts. However, Sedimenticolaceae itself is also not exclusively a family of symbiotic bacteria. There are at least two isolates within this family that are not known to occur in animal hosts (*Sedimenticola thiotaurini* and *Sedimenticola selenatireducans*). This MAG’s low abundance compared with the Thiohalomonadales species in metagenomes and amplicon libraries of one *B. thionipta* individual, and its absence from all metagenomes and amplicon libraries of the other four individuals would seem to indicate that if it is a symbiont, it is rare. It is tempting to speculate that this MAG may represent a symbiont from *B. thionipta* that was replaced by the novel Thiohalomonadales symbiont, but without additional individuals and extensive analysis, we

cannot be sure. Considering these uncertainties, we did not include it in further metabolic analyses.

Naming of *Ca.* T. gloverae

Ca. T. gloverae was named after Emily Glover, associated with the Natural History Museum, London, UK, whose decades of research and work on Lucinidae taxonomy and phylogeny have enabled this research.

Lucinoma aequizonata symbionts show reduction in genome size and metabolic capabilities

All seven *Lucinoma aequizonata* samples within this study (seven) produced single MAGs assigned to *Ca.* Thiodiazotropha and grouped into a single species group, Aeq1. The average genome size of this species group (3.03 megabases) was well below the average of all other *Ca.* Thiodiazotropha MAGs (Supplemental Results). This was also coupled with reduced metabolic capabilities (Dataset S5 and S7), such as a lack of denitrification and flagellar genes. While these MAGs are not circularized (Dataset S3), the high sample number (n=7), both from fresh and preserved *Lucinoma aequizonata*, alongside these results suggests a sign of genome reduction and is commonly related to Muller's ratchet[8]. Genome reduction is commonly associated with vertical transmission and the associated population bottlenecks. This alone suggests that these symbionts might be acquired vertically, however, all lucinids studied to date are considered to acquire symbionts horizontally[9].

The site where *Lucinoma aequizonata* are found (Santa Barbara Basin, California, USA), which is an oxygen minimum zone[10,11] (OMZ). The unique site that these host samples were collected

from is also inherently restricted in resources for a typical lucinid symbiosis (both oxygen and sulfur compounds are primary components). Sediments within the basin are anoxic within millimeters of the surface[10], leading to metabolic adjustments by organisms living there. This is likely not an artifact of assembly as all Aeq1 MAGs display consistent metabolic functionality, and only have a single variation in metabolism among all MAGs (Dataset S7). *Lucinoma aequizonata* has been documented surviving 262 days[12] at the expense of its symbiont population which respires nitrate as an alternative to oxygen under such conditions[10,11,13], a capability that was found in all Aeq1 MAGs (Supplemental Results). These MAGs also lack the genes needed for a flagellar assembly, suggesting a lack of motility. While motility genes have been found to be expressed in gills[14], it has been speculated that motility is vital for the environmental stage of the symbiont life cycle and infection of lucinid gills, similar to infectious bacteria[15]. Therefore, the lack of motility in addition to the likely genome reduction of Aeq1 species genomes raises questions about the *Lucinoma aequizonata*-symbiont relationship. This symbiont species possesses the basic qualification of being an obligately host-associated symbiont.

SI Figures and Tables

Figure S1

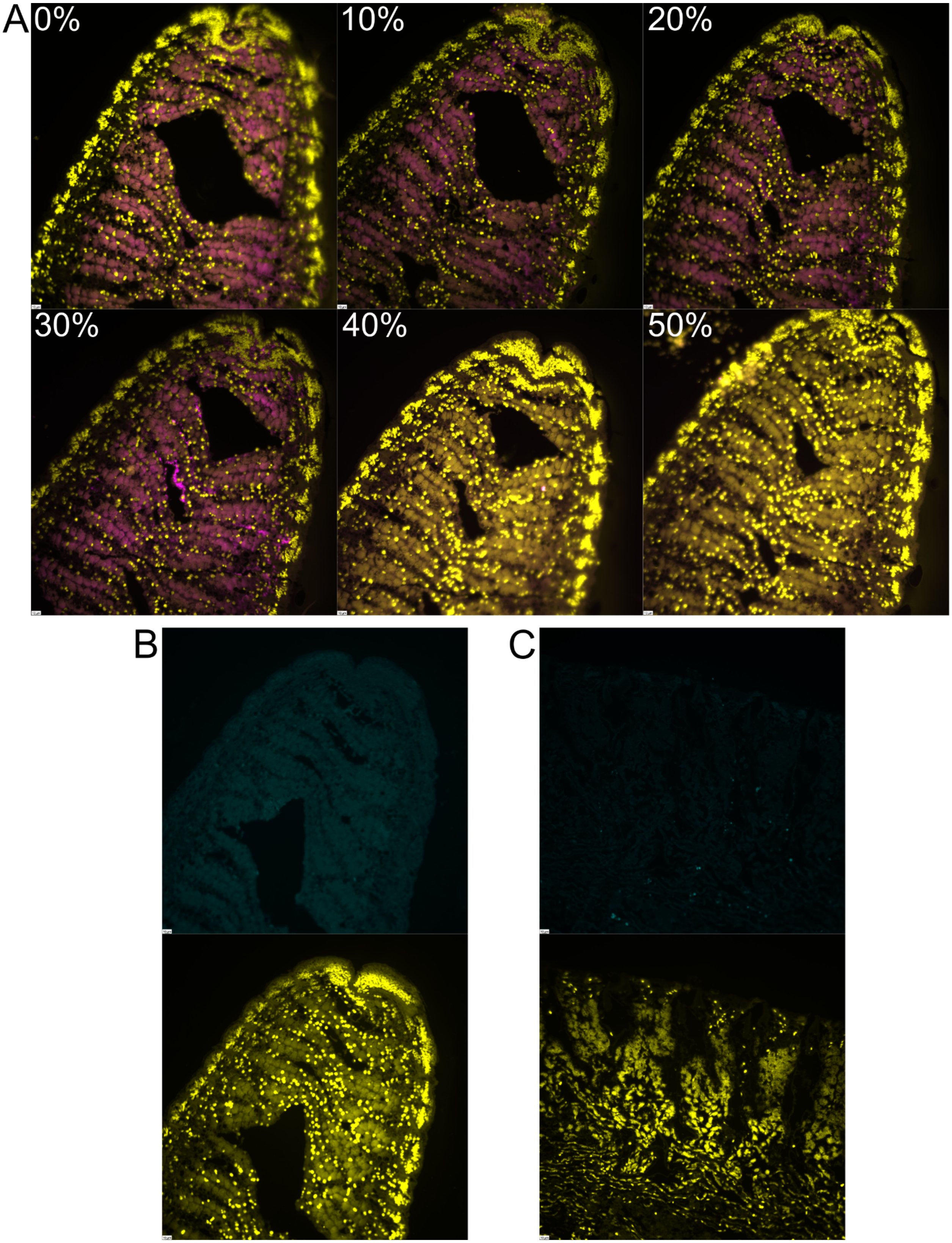
A
0%
10%
20%
30%
40%
50%
B
C

Figure S1: A) CARD-FISH formamide series using sample L130 (*Lucinoma myriamae*) to determine the optimal binding concentration. Percentages displayed represent the formamide concentration tested. DAPI = yellow and CARD-FISH probe = purple. B) non-EUB probe tests from *Lucinoma myriamae*. DAPI = yellow and non-EUB probe = purple. C) non-EUB probe tests from *Bathyaustriella thionipta*. DAPI = yellow and non-EUB probe = purple

Figure S2

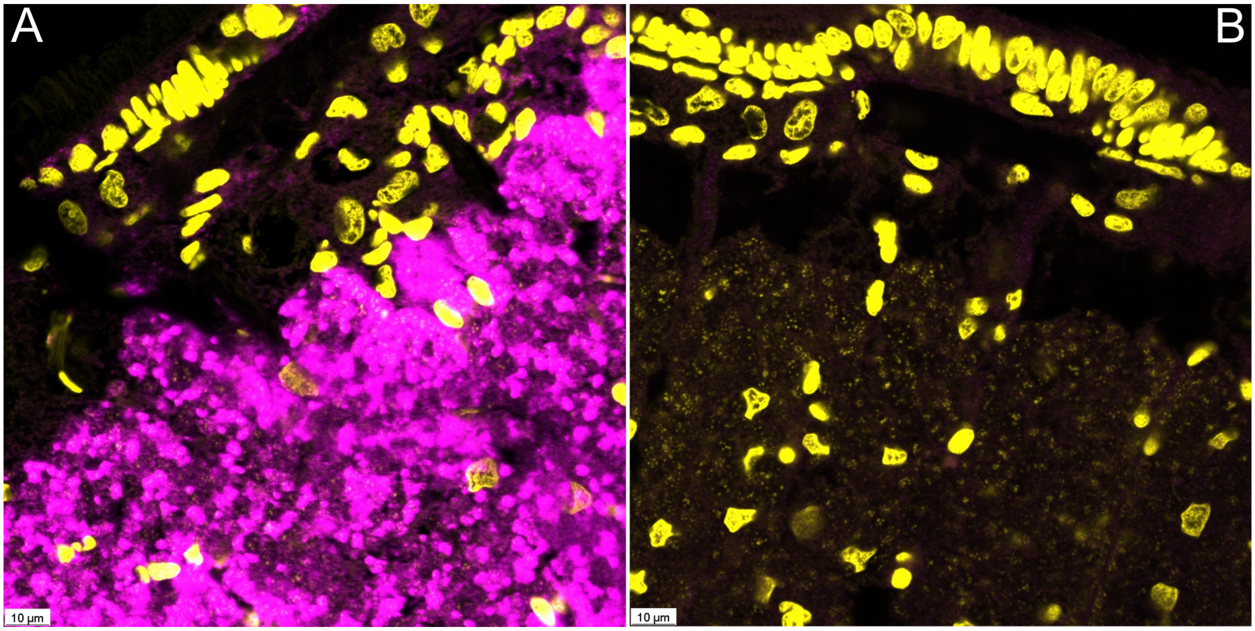

Figure S2: CARD-FISH specificity test of thiohalomonadales-specific probe. A) Sample L130 (*Lucinoma myriamae*) gill sample. B) *Loripes orbiculatus* gill sample. DAPI = yellow, thiohalomonadales-specific probe = purple. No binding to *Ca.* Thiodiazotropha bacteria in image B was found, suggesting that this probe is specific to Thiohalomonadales species.

Figure S3

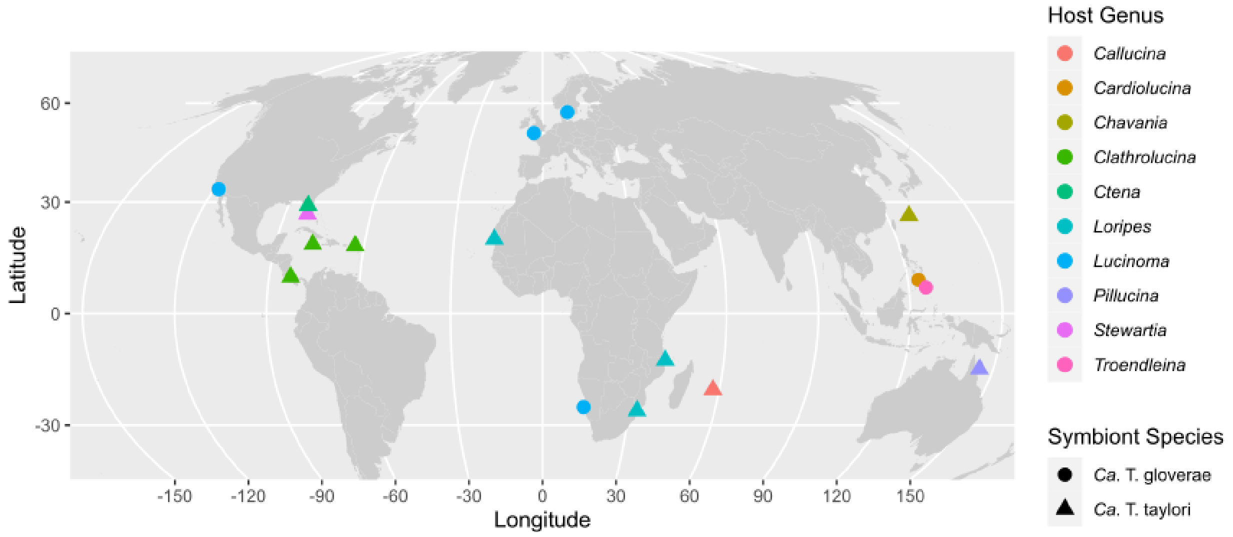

Figure S3: Geographical distribution of samples containing *Ca.* T. gloverae and *Ca.* T. taylori, the two symbiont species with panglobal distributions.

Figure S4

A)

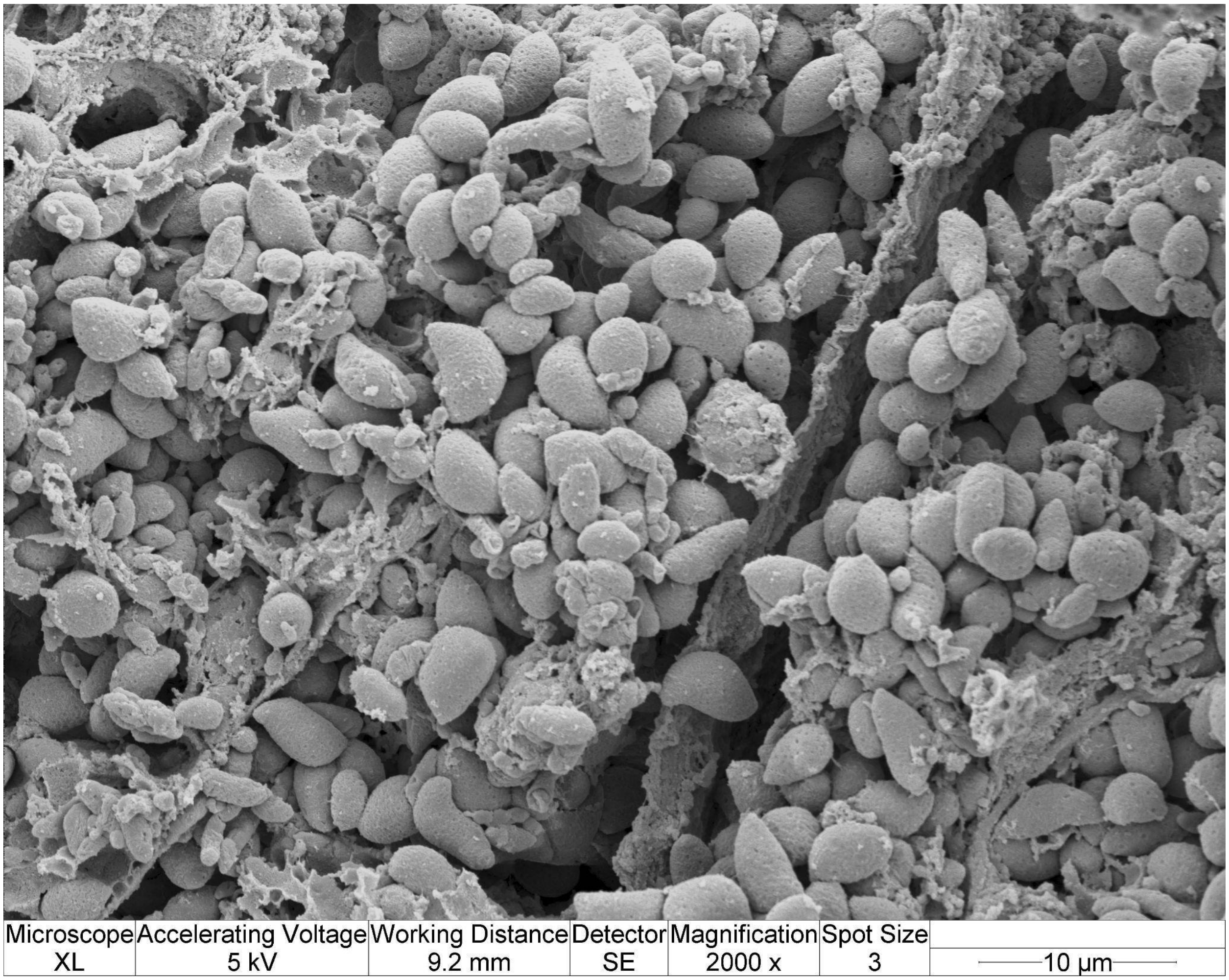
Microscope
XL
Accelerating Voltage
5 kV
Working Distance
9.2 mm
Detector
SE
Magnification
2000 x
Spot Size
3
10 µm

B)

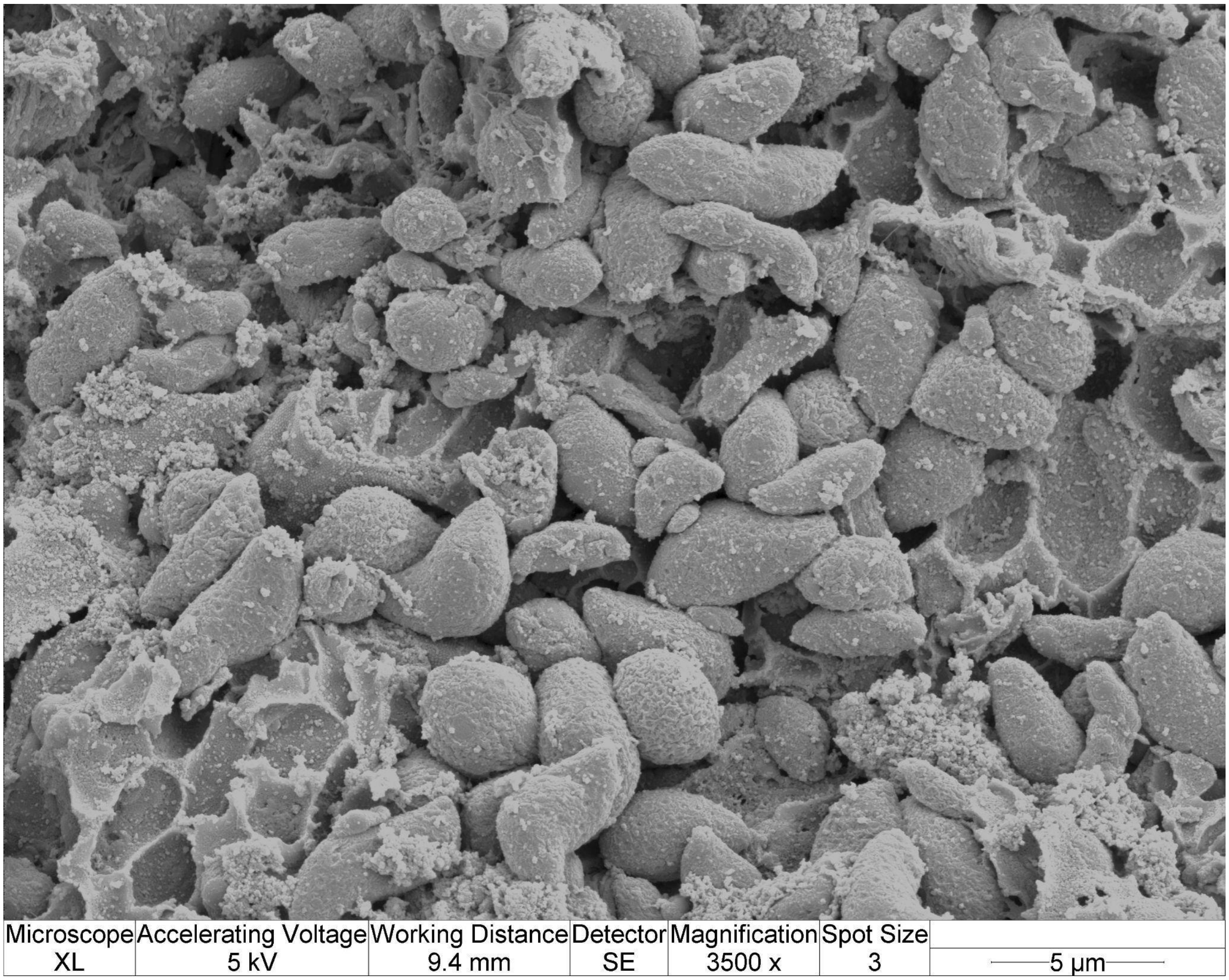
Microscope
Accelerating Voltage
Working Distance
Detector
Magnification
Spot Size
XL
5 kV
9.4 mm
SE
3500 x
3
5 μm

C)

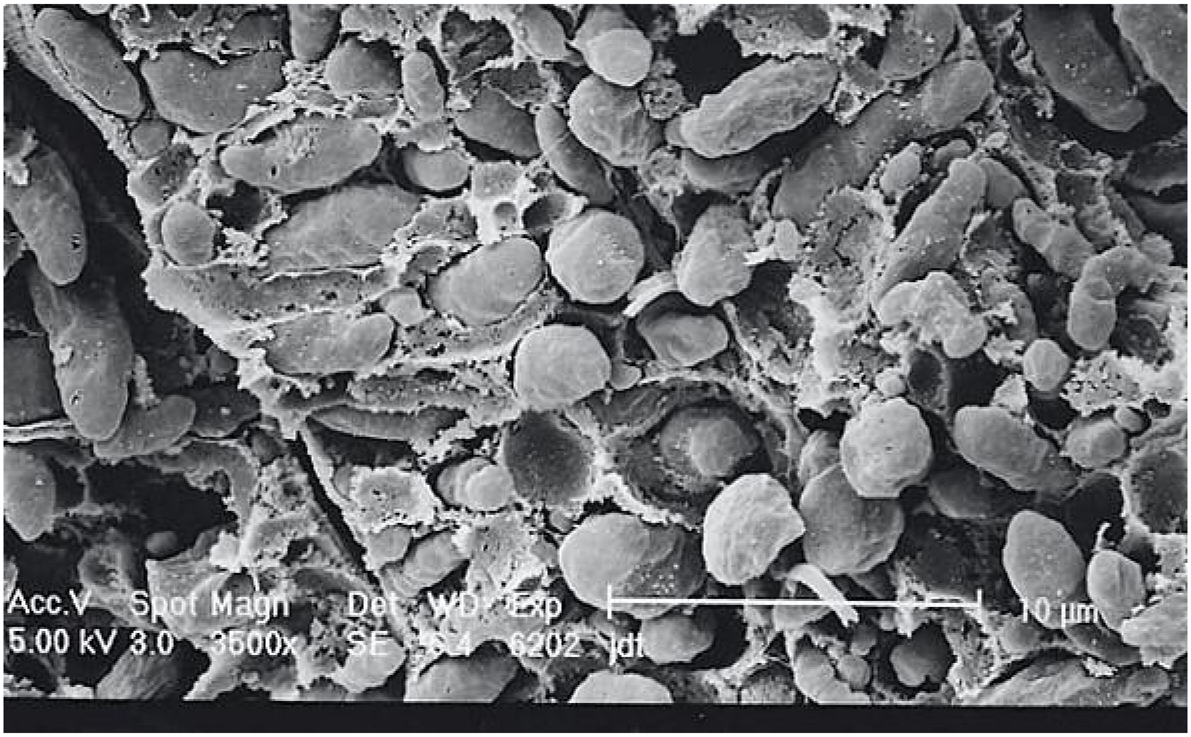
Acc.V Spot Magn Det WD Exp
5.00 kV 3.0 3500x SE 6202 jdt
10 µm

D)

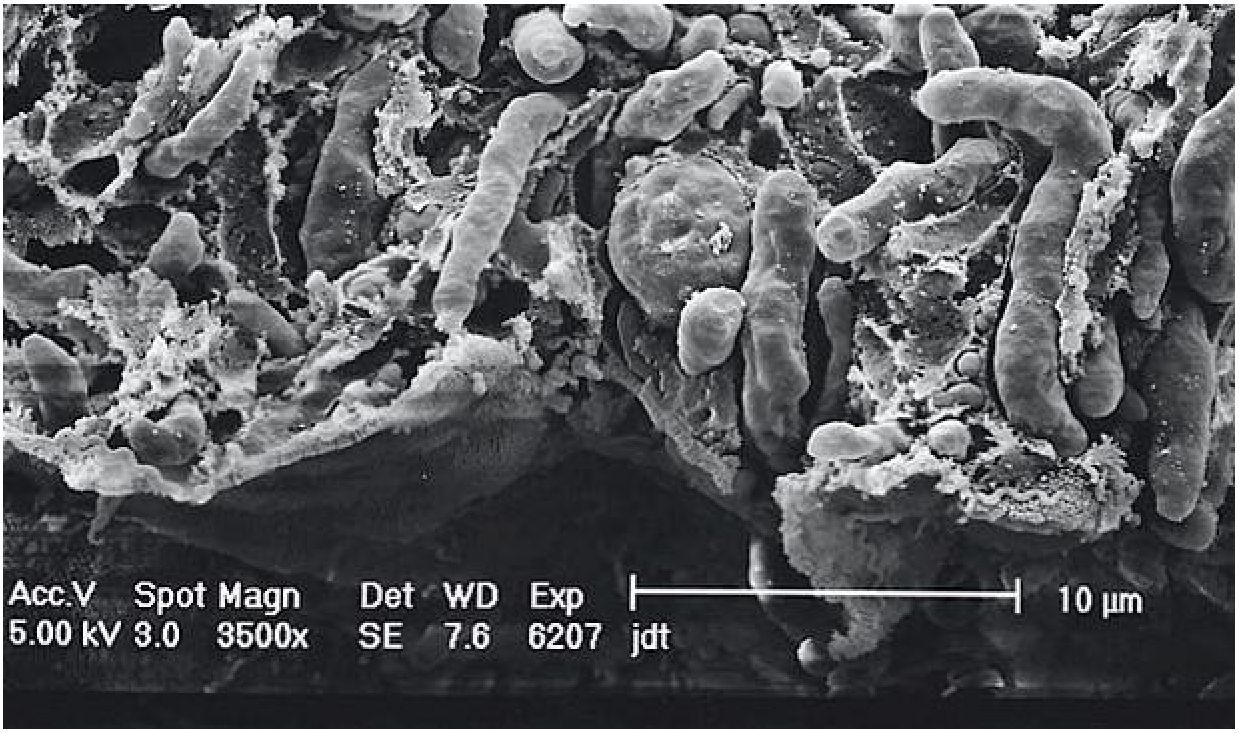
Acc.V Spot Magn Det WD Exp
5.00 kV 3.0 3500x SE 7.6 6207 jdt
10 µm

E)

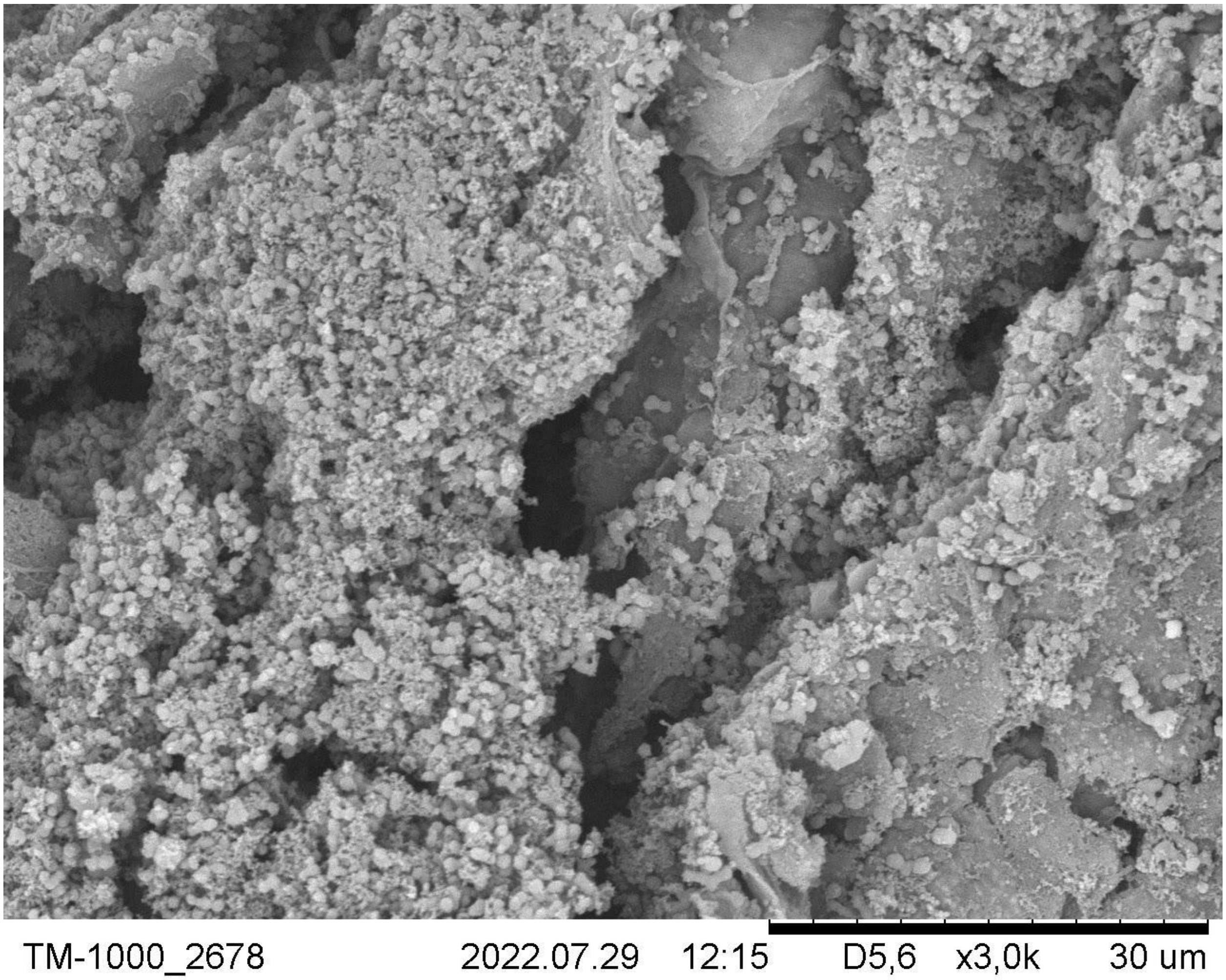

F)

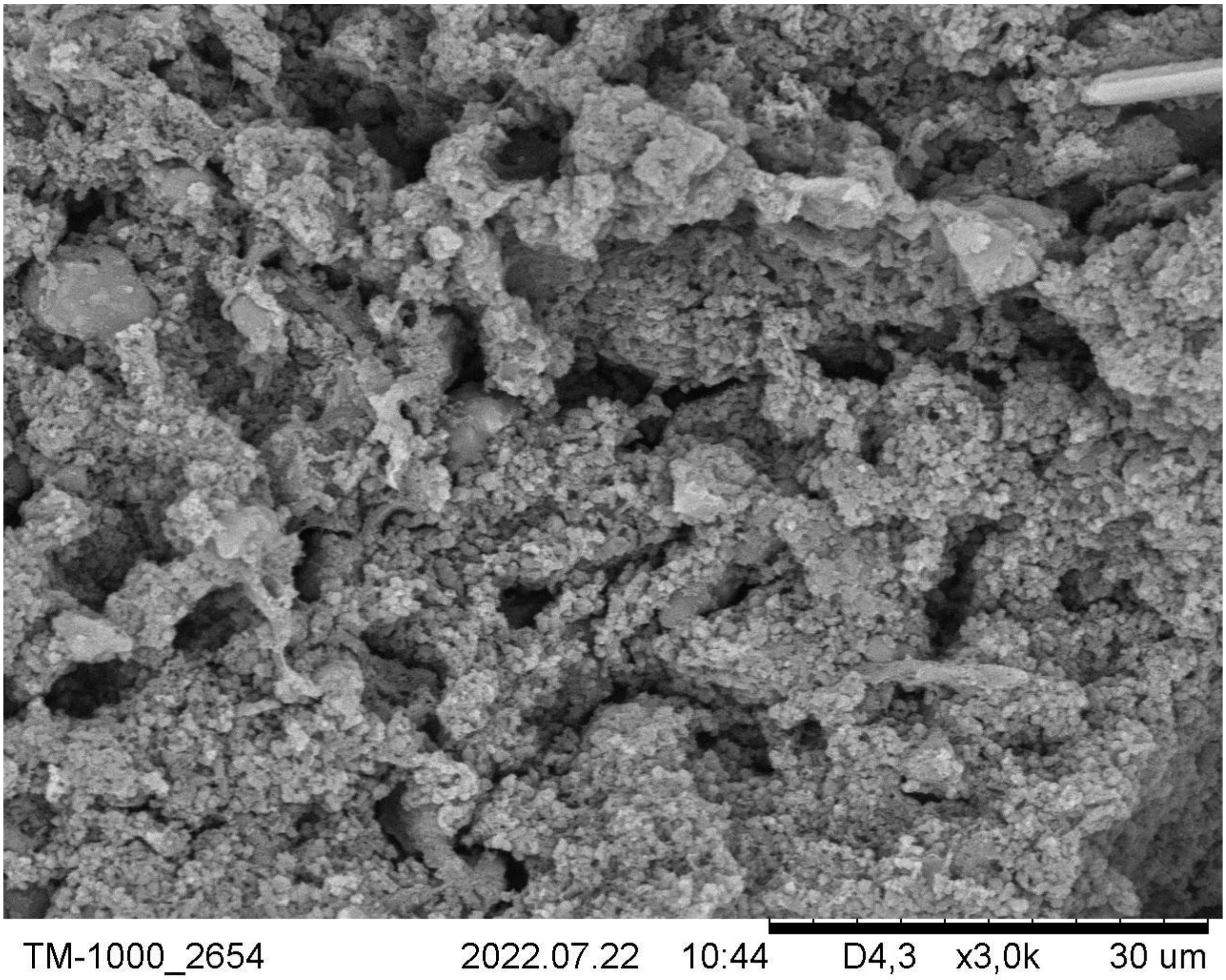

Figure S4: Scanning electron microscopy shows that various lucinid species host intracellular gill symbionts. A) *L. borealis* bacteriocyte showing bacteria; B) *L. borealis* bacteriocyte showing bacteria; C) gill filament of *Rugalucina munda* showing bacteria D) gill filament of *Rugalucina munda* showing bacteria. E) gill filament of *Bathyaustriella thionipta* showing bacteria. Also shown in Glover et. al. 2004 [5]. F) gill filament of *Lucinoma myriamae* showing bacteria.

Figure S5

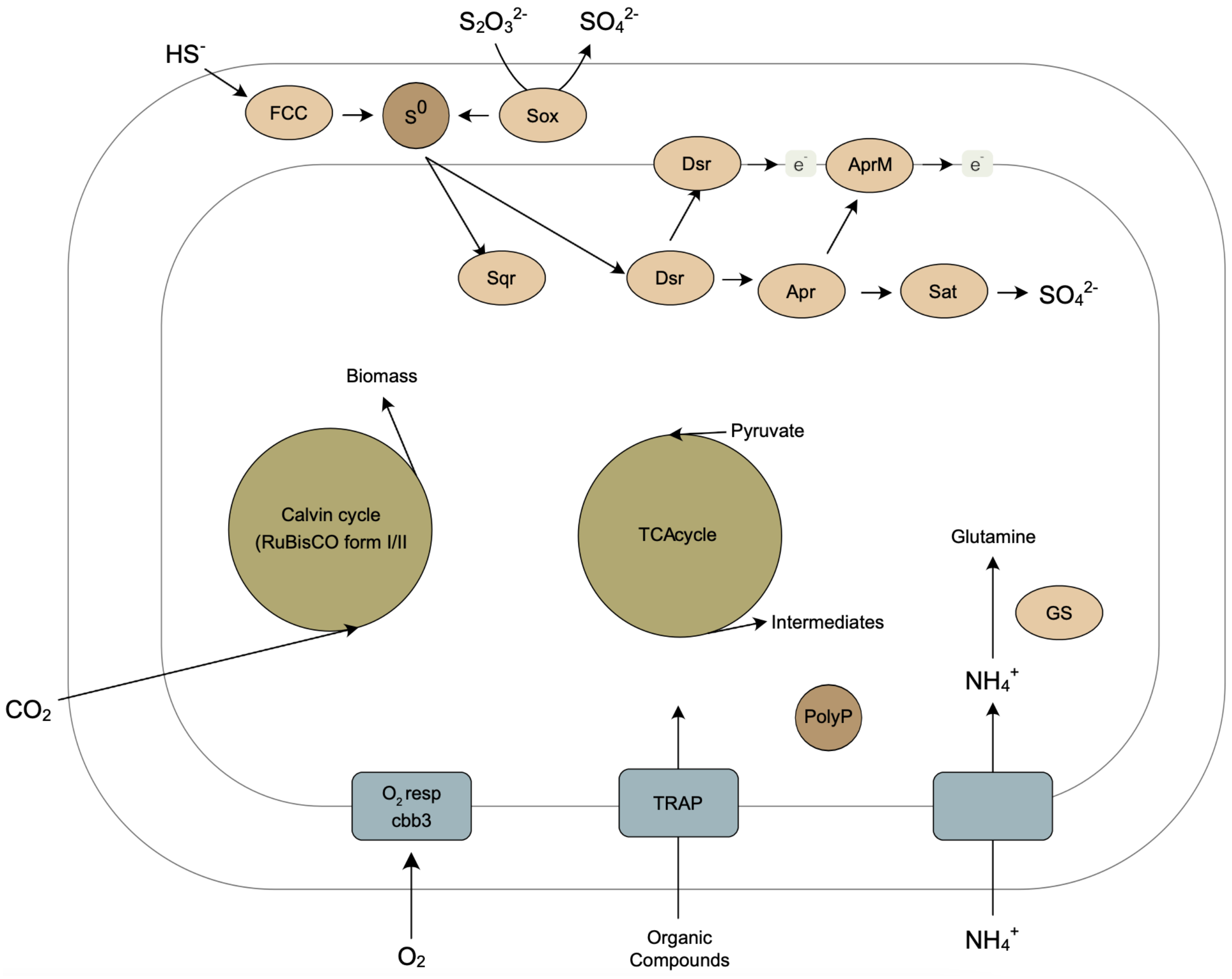

Figure S5. Carbon fixation, sulfur oxidation, and ammonia assimilation are amongst the core metabolic functions shared by lucinid symbionts from the *Ca*. Thiodiazotropha, Sedimenticola and Thiohalomonadales lineages. Extensive metabolic summaries can be found in **Table S5 and S6.** IM, inner membrane; OM, outer membrane; HPP, proton-translocating pyrophosphatase; FCC, flavocytochrome c; S^0, elemental sulfur granule; Sox, sox enzyme system for sulfur oxidation; Dsr, reverse dissimilatory sulfite reductase; Apr, adenosine phosphosulfate reductase; AprM, adenosine 5′-phosphosulfate membrane anchor; Sat, sulfate adenylyltransferase; O^2 resp, genes for respiring oxygen (cbb3-type cytochrome c oxidase); TRAP, TRAP transporter; PolyP, polyphosphate granule; GlnA, glutamine synthetase

Figure S6

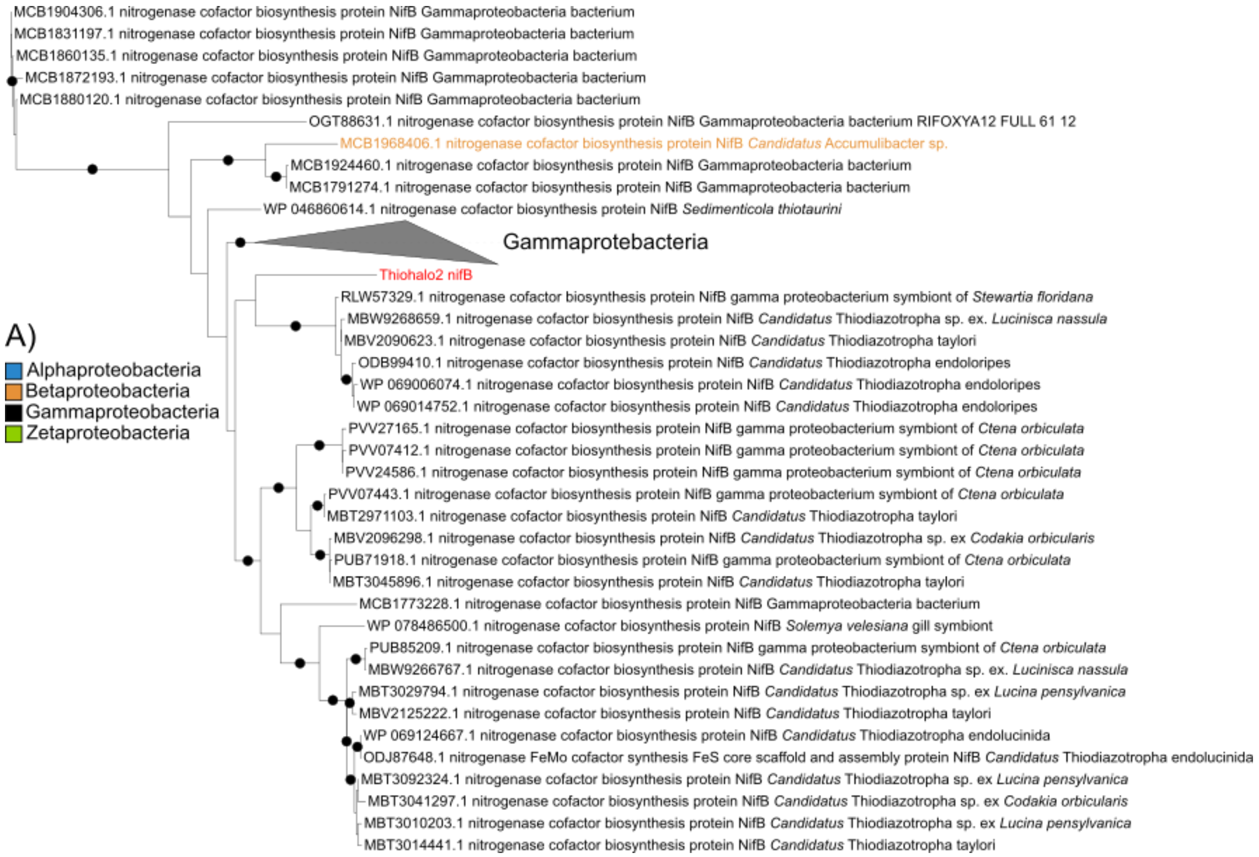
MCB1904306.1 nitrogenase cofactor biosynthesis protein NifB Gammaproteobacteria bacterium
MCB1831197.1 nitrogenase cofactor biosynthesis protein NifB Gammaproteobacteria bacterium
MCB1860135.1 nitrogenase cofactor biosynthesis protein NifB Gammaproteobacteria bacterium
MCB1872193.1 nitrogenase cofactor biosynthesis protein NifB Gammaproteobacteria bacterium
MCB1880120.1 nitrogenase cofactor biosynthesis protein NifB Gammaproteobacteria bacterium
OGT88631.1 nitrogenase cofactor biosynthesis protein NifB Gammaproteobacteria bacterium RIFOXYA12 FULL 61 12
MCB1968406.1 nitrogenase cofactor biosynthesis protein NifB Candidatus Accumulibacter sp.
MCB1924460.1 nitrogenase cofactor biosynthesis protein NifB Gammaproteobacteria bacterium
MCB1791274.1 nitrogenase cofactor biosynthesis protein NifB Gammaproteobacteria bacterium
WP 046860614.1 nitrogenase cofactor biosynthesis protein NifB Sedimenticola thiotaurini
Gammaprotebacteria
Thiohalo2 nifB
RLW57329.1 nitrogenase cofactor biosynthesis protein NifB gamma proteobacterium symbiont of Stewartia floridana
MBW9268659.1 nitrogenase cofactor biosynthesis protein NifB Candidatus Thiodiazotropha sp. ex. Lucinisca nassula
MBV2090623.1 nitrogenase cofactor biosynthesis protein NifB Candidatus Thiodiazotropha taylori
ODB99410.1 nitrogenase cofactor biosynthesis protein NifB Candidatus Thiodiazotropha endoloripes
WP 069006074.1 nitrogenase cofactor biosynthesis protein NifB Candidatus Thiodiazotropha endoloripes
WP 069014752.1 nitrogenase cofactor biosynthesis protein NifB Candidatus Thiodiazotropha endoloripes
PVV27165.1 nitrogenase cofactor biosynthesis protein NifB gamma proteobacterium symbiont of Ctena orbiculata
PVV07412.1 nitrogenase cofactor biosynthesis protein NifB gamma proteobacterium symbiont of Ctena orbiculata
PVV24586.1 nitrogenase cofactor biosynthesis protein NifB gamma proteobacterium symbiont of Ctena orbiculata
PVV07443.1 nitrogenase cofactor biosynthesis protein NifB gamma proteobacterium symbiont of Ctena orbiculata
MBT2971103.1 nitrogenase cofactor biosynthesis protein NifB Candidatus Thiodiazotropha taylori
MBV2096298.1 nitrogenase cofactor biosynthesis protein NifB Candidatus Thiodiazotropha sp. ex Codakia orbicularis
PUB71918.1 nitrogenase cofactor biosynthesis protein NifB gamma proteobacterium symbiont of Ctena orbiculata
MBT3045896.1 nitrogenase cofactor biosynthesis protein NifB Candidatus Thiodiazotropha taylori
MCB1773228.1 nitrogenase cofactor biosynthesis protein NifB Gammaproteobacteria bacterium
WP 078486500.1 nitrogenase cofactor biosynthesis protein NifB Solemya velesiana gill symbiont
PUB85209.1 nitrogenase cofactor biosynthesis protein NifB gamma proteobacterium symbiont of Ctena orbiculata
MBW9266767.1 nitrogenase cofactor biosynthesis protein NifB Candidatus Thiodiazotropha sp. ex. Lucinisca nassula
MBT3029794.1 nitrogenase cofactor biosynthesis protein NifB Candidatus Thiodiazotropha sp. ex Lucina pensylvanica
MBV2125222.1 nitrogenase cofactor biosynthesis protein NifB Candidatus Thiodiazotropha taylori
WP 069124667.1 nitrogenase cofactor biosynthesis protein NifB Candidatus Thiodiazotropha endolucinida
ODJ87648.1 nitrogenase FeMo cofactor synthesis FeS core scaffold and assembly protein NifB Candidatus Thiodiazotropha endolucinida
MBT3092324.1 nitrogenase cofactor biosynthesis protein NifB Candidatus Thiodiazotropha sp. ex Lucina pensylvanica
MBT3041297.1 nitrogenase cofactor biosynthesis protein NifB Candidatus Thiodiazotropha sp. ex Codakia orbicularis
MBT3010203.1 nitrogenase cofactor biosynthesis protein NifB Candidatus Thiodiazotropha sp. ex Lucina pensylvanica
MBT3014441.1 nitrogenase cofactor biosynthesis protein NifB Candidatus Thiodiazotropha taylori
A)
Alphaproteobacteria
Betaproteobacteria
Gammaproteobacteria
Zetaproteobacteria

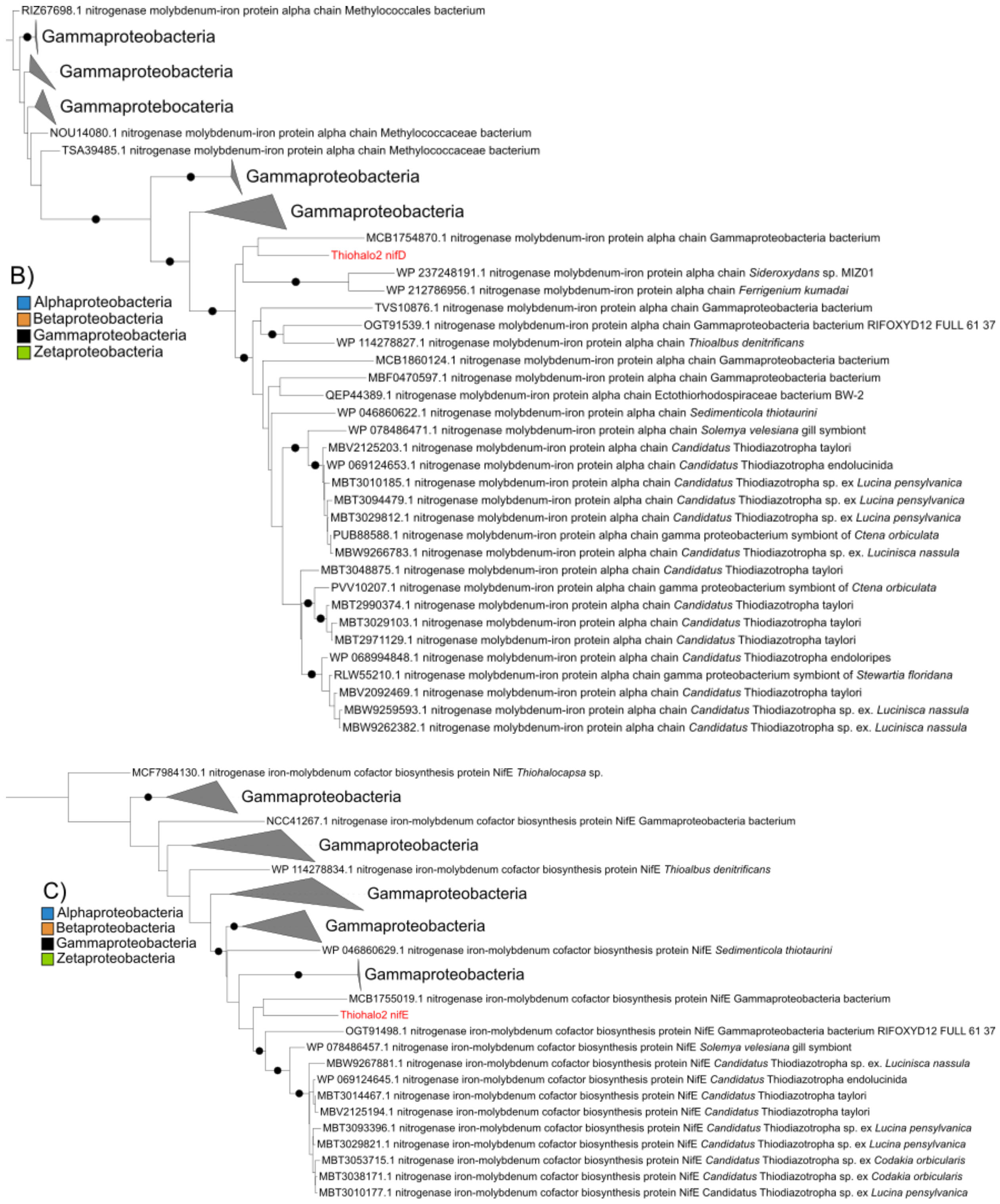

RIZ67698.1 nitrogenase molybdenum-iron protein alpha chain Methylococcales bacterium
Gammaproteobacteria
Gammaproteobacteria
Gammaprotebocateria
NOU14080.1 nitrogenase molybdenum-iron protein alpha chain Methylococcaceae bacterium
TSA39485.1 nitrogenase molybdenum-iron protein alpha chain Methylococcaceae bacterium
Gammaproteobacteria
Gammaproteobacteria
MCB1754870.1 nitrogenase molybdenum-iron protein alpha chain Gammaproteobacteria bacterium
Thiohalo2 nifD
B)
Alphaproteobacteria
Betaproteobacteria
Gammaproteobacteria
Zetaproteobacteria
WP 237248191.1 nitrogenase molybdenum-iron protein alpha chain Sideroxydans sp. MIZ01
WP 212786956.1 nitrogenase molybdenum-iron protein alpha chain Ferrigenium kumadai
TVS10876.1 nitrogenase molybdenum-iron protein alpha chain Gammaproteobacteria bacterium
OGT91539.1 nitrogenase molybdenum-iron protein alpha chain Gammaproteobacteria bacterium RIFOXYD12 FULL 61 37
WP 114278827.1 nitrogenase molybdenum-iron protein alpha chain Thioalbus denitrificans
MCB1860124.1 nitrogenase molybdenum-iron protein alpha chain Gammaproteobacteria bacterium
MBF0470597.1 nitrogenase molybdenum-iron protein alpha chain Gammaproteobacteria bacterium
QEP44389.1 nitrogenase molybdenum-iron protein alpha chain Ectothiorhodospiraceae bacterium BW-2
WP 046860622.1 nitrogenase molybdenum-iron protein alpha chain Sedimenticola thiotaurini
WP 078486471.1 nitrogenase molybdenum-iron protein alpha chain Solemya velesiana gill symbiont
MBV2125203.1 nitrogenase molybdenum-iron protein alpha chain Candidatus Thiodiazotropha taylori
WP 069124653.1 nitrogenase molybdenum-iron protein alpha chain Candidatus Thiodiazotropha endolucinida
MBT3010185.1 nitrogenase molybdenum-iron protein alpha chain Candidatus Thiodiazotropha sp. ex Lucina pensylvanica
MBT3094479.1 nitrogenase molybdenum-iron protein alpha chain Candidatus Thiodiazotropha sp. ex Lucina pensylvanica
MBT3029812.1 nitrogenase molybdenum-iron protein alpha chain Candidatus Thiodiazotropha sp. ex Lucina pensylvanica
PUB88588.1 nitrogenase molybdenum-iron protein alpha chain gamma proteobacterium symbiont of Ctena orbiculata
MBW9266783.1 nitrogenase molybdenum-iron protein alpha chain Candidatus Thiodiazotropha sp. ex. Lucinisca nassula
MBT3048875.1 nitrogenase molybdenum-iron protein alpha chain Candidatus Thiodiazotropha taylori
PVV10207.1 nitrogenase molybdenum-iron protein alpha chain gamma proteobacterium symbiont of Ctena orbiculata
MBT2990374.1 nitrogenase molybdenum-iron protein alpha chain Candidatus Thiodiazotropha taylori
MBT3029103.1 nitrogenase molybdenum-iron protein alpha chain Candidatus Thiodiazotropha taylori
MBT2971129.1 nitrogenase molybdenum-iron protein alpha chain Candidatus Thiodiazotropha taylori
WP 068994848.1 nitrogenase molybdenum-iron protein alpha chain Candidatus Thiodiazotropha endoloripes
RLW55210.1 nitrogenase molybdenum-iron protein alpha chain gamma proteobacterium symbiont of Stewartia floridana
MBV2092469.1 nitrogenase molybdenum-iron protein alpha chain Candidatus Thiodiazotropha taylori
MBW9259593.1 nitrogenase molybdenum-iron protein alpha chain Candidatus Thiodiazotropha sp. ex. Lucinisca nassula
MBW9262382.1 nitrogenase molybdenum-iron protein alpha chain Candidatus Thiodiazotropha sp. ex. Lucinisca nassula
MCF7984130.1 nitrogenase iron-molybdenum cofactor biosynthesis protein NifE Thiohalocapsa sp.
Gammaproteobacteria
NCC41267.1 nitrogenase iron-molybdenum cofactor biosynthesis protein NifE Gammaproteobacteria bacterium
Gammaproteobacteria
WP 114278834.1 nitrogenase iron-molybdenum cofactor biosynthesis protein NifE Thioalbus denitrificans
C)
Alphaproteobacteria
Betaproteobacteria
Gammaproteobacteria
Zetaproteobacteria
Gammaproteobacteria
Gammaproteobacteria
WP 046860629.1 nitrogenase iron-molybdenum cofactor biosynthesis protein NifE Sedimenticola thiotaurini
Gammaproteobacteria
MCB1755019.1 nitrogenase iron-molybdenum cofactor biosynthesis protein NifE Gammaproteobacteria bacterium
Thiohalo2 nifE
OGT91498.1 nitrogenase iron-molybdenum cofactor biosynthesis protein NifE Gammaproteobacteria bacterium RIFOXYD12 FULL 61 37
WP 078486457.1 nitrogenase iron-molybdenum cofactor biosynthesis protein NifE Solemya velesiana gill symbiont
MBW9267881.1 nitrogenase iron-molybdenum cofactor biosynthesis protein NifE Candidatus Thiodiazotropha sp. ex. Lucinisca nassula
WP 069124645.1 nitrogenase iron-molybdenum cofactor biosynthesis protein NifE Candidatus Thiodiazotropha endolucinida
MBT3014467.1 nitrogenase iron-molybdenum cofactor biosynthesis protein NifE Candidatus Thiodiazotropha taylori
MBV2125194.1 nitrogenase iron-molybdenum cofactor biosynthesis protein NifE Candidatus Thiodiazotropha taylori
MBT3093396.1 nitrogenase iron-molybdenum cofactor biosynthesis protein NifE Candidatus Thiodiazotropha sp. ex Lucina pensylvanica
MBT3029821.1 nitrogenase iron-molybdenum cofactor biosynthesis protein NifE Candidatus Thiodiazotropha sp. ex Lucina pensylvanica
MBT3053715.1 nitrogenase iron-molybdenum cofactor biosynthesis protein NifE Candidatus Thiodiazotropha sp. ex Codakia orbicularis
MBT3038171.1 nitrogenase iron-molybdenum cofactor biosynthesis protein NifE Candidatus Thiodiazotropha sp. ex Codakia orbicularis
MBT3010177.1 nitrogenase iron-molybdenum cofactor biosynthesis protein NifE Candidatus Thiodiazotropha sp. ex Lucina pensylvanica

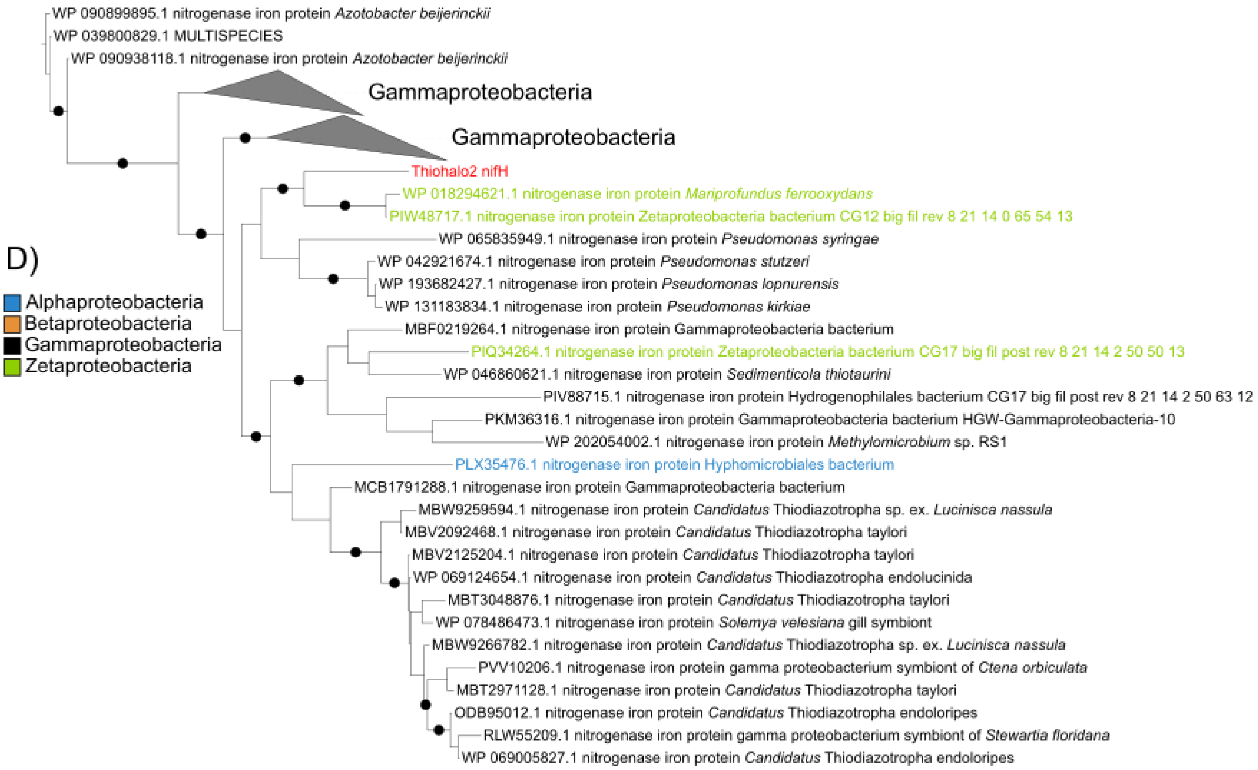
D)
Alphaproteobacteria
Betaproteobacteria
Gammaproteobacteria
Zetaproteobacteria
WP 090899895.1 nitrogenase iron protein Azotobacter beijerinckii
WP 039800829.1 MULTISPECIES
WP 090938118.1 nitrogenase iron protein Azotobacter beijerinckii
Gammaproteobacteria
Gammaproteobacteria
Thiohalo2 nifH
WP 018294621.1 nitrogenase iron protein Mariprofundus ferrooxydans
PIW48717.1 nitrogenase iron protein Zetaproteobacteria bacterium CG12 big fil rev 8 21 14 0 65 54 13
WP 065835949.1 nitrogenase iron protein Pseudomonas syringae
WP 042921674.1 nitrogenase iron protein Pseudomonas stutzeri
WP 193682427.1 nitrogenase iron protein Pseudomonas lopnurensis
WP 131183834.1 nitrogenase iron protein Pseudomonas kirkiae
MBF0219264.1 nitrogenase iron protein Gammaproteobacteria bacterium
PIQ34264.1 nitrogenase iron protein Zetaproteobacteria bacterium CG17 big fil post rev 8 21 14 2 50 50 13
WP 046860621.1 nitrogenase iron protein Sedimenticola thiotaurini
PIV88715.1 nitrogenase iron protein Hydrogenophilales bacterium CG17 big fil post rev 8 21 14 2 50 63 12
PKM36316.1 nitrogenase iron protein Gammaproteobacteria bacterium HGW-Gammaproteobacteria-10
WP 202054002.1 nitrogenase iron protein Methylomicrobium sp. RS1
PLX35476.1 nitrogenase iron protein Hyphomicrobiales bacterium
MCB1791288.1 nitrogenase iron protein Gammaproteobacteria bacterium
MBW9259594.1 nitrogenase iron protein Candidatus Thiodiazotropha sp. ex. Lucinisca nassula
MBV2092468.1 nitrogenase iron protein Candidatus Thiodiazotropha taylori
MBV2125204.1 nitrogenase iron protein Candidatus Thiodiazotropha taylori
WP 069124654.1 nitrogenase iron protein Candidatus Thiodiazotropha endolucinida
MBT3048876.1 nitrogenase iron protein Candidatus Thiodiazotropha taylori
WP 078486473.1 nitrogenase iron protein Solemya velesiana gill symbiont
MBW9266782.1 nitrogenase iron protein Candidatus Thiodiazotropha sp. ex. Lucinisca nassula
PVV10206.1 nitrogenase iron protein gamma proteobacterium symbiont of Ctena orbiculata
MBT2971128.1 nitrogenase iron protein Candidatus Thiodiazotropha taylori
ODB95012.1 nitrogenase iron protein Candidatus Thiodiazotropha endoloripes
RLW55209.1 nitrogenase iron protein gamma proteobacterium symbiont of Stewartia floridana
WP 069005827.1 nitrogenase iron protein Candidatus Thiodiazotropha endoloripes

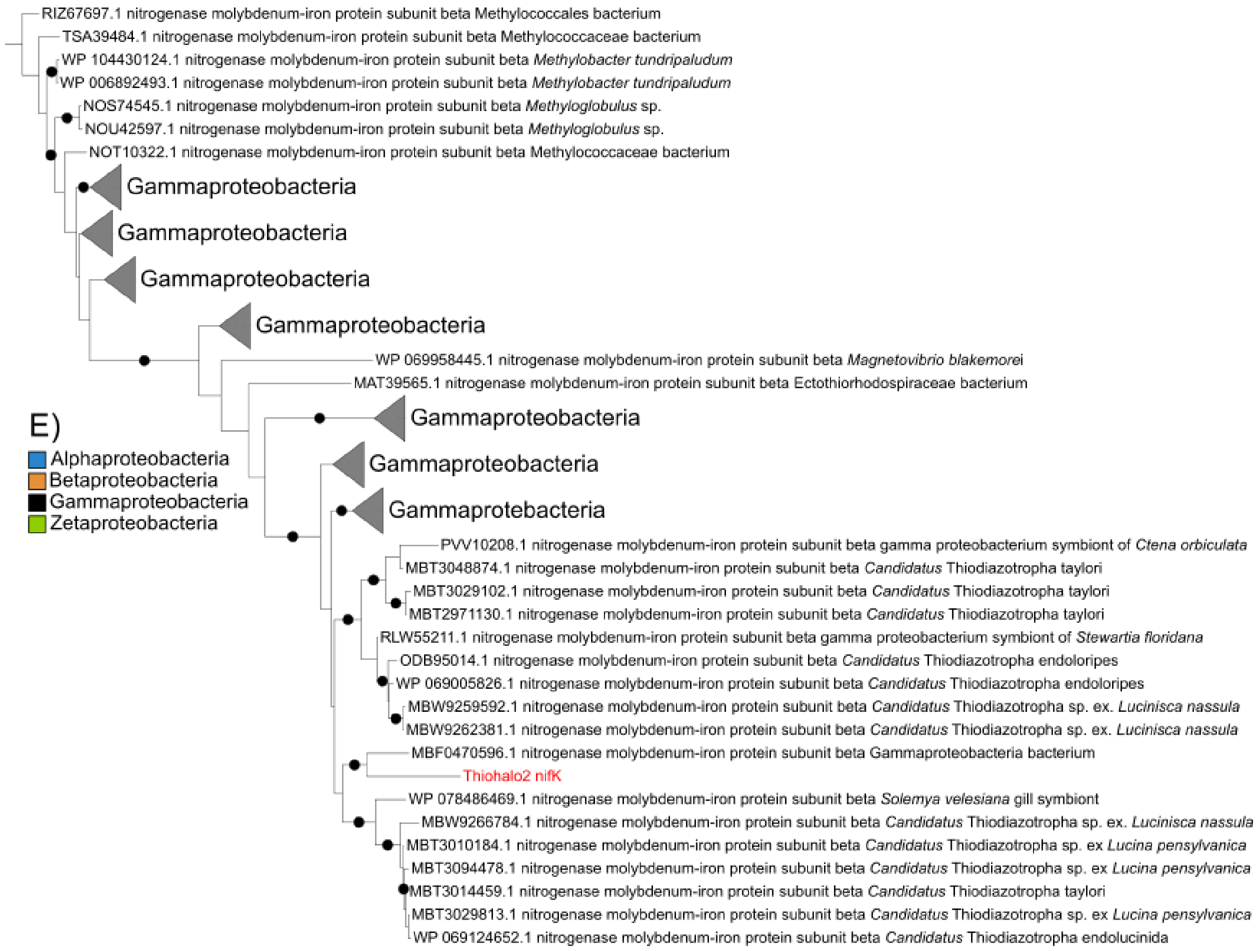
RIZ67697.1 nitrogenase molybdenum-iron protein subunit beta Methylococcales bacterium
TSA39484.1 nitrogenase molybdenum-iron protein subunit beta Methylococcaceae bacterium
WP 104430124.1 nitrogenase molybdenum-iron protein subunit beta *Methylobacter tundripaludum*
WP 006892493.1 nitrogenase molybdenum-iron protein subunit beta *Methylobacter tundripaludum*
NOS74545.1 nitrogenase molybdenum-iron protein subunit beta *Methyloglobulus* sp.
NOU42597.1 nitrogenase molybdenum-iron protein subunit beta *Methyloglobulus* sp.
NOT10322.1 nitrogenase molybdenum-iron protein subunit beta Methylococcaceae bacterium
Gammaproteobacteria
Gammaproteobacteria
Gammaproteobacteria
Gammaproteobacteria
WP 069958445.1 nitrogenase molybdenum-iron protein subunit beta *Magnetovibrio blakemorei*
MAT39565.1 nitrogenase molybdenum-iron protein subunit beta Ectothiorhodospiraceae bacterium
E)
Alphaproteobacteria
Betaproteobacteria
Gammaproteobacteria
Zetaproteobacteria
Gammaproteobacteria
Gammaproteobacteria
Gammaprotebacteria
PVV10208.1 nitrogenase molybdenum-iron protein subunit beta gamma proteobacterium symbiont of *Ctena orbiculata*
MBT3048874.1 nitrogenase molybdenum-iron protein subunit beta *Candidatus* Thiodiazotropha taylori
MBT3029102.1 nitrogenase molybdenum-iron protein subunit beta *Candidatus* Thiodiazotropha taylori
MBT2971130.1 nitrogenase molybdenum-iron protein subunit beta *Candidatus* Thiodiazotropha taylori
RLW55211.1 nitrogenase molybdenum-iron protein subunit beta gamma proteobacterium symbiont of *Stewartia floridana*
ODB95014.1 nitrogenase molybdenum-iron protein subunit beta *Candidatus* Thiodiazotropha endoloripes
WP 069005826.1 nitrogenase molybdenum-iron protein subunit beta *Candidatus* Thiodiazotropha endoloripes
MBW9259592.1 nitrogenase molybdenum-iron protein subunit beta *Candidatus* Thiodiazotropha sp. ex. *Lucinisca nassula*
MBW9262381.1 nitrogenase molybdenum-iron protein subunit beta *Candidatus* Thiodiazotropha sp. ex. *Lucinisca nassula*
MBF0470596.1 nitrogenase molybdenum-iron protein subunit beta Gammaproteobacteria bacterium
Thiohalo2 nifK
WP 078486469.1 nitrogenase molybdenum-iron protein subunit beta *Solemya velesiana* gill symbiont
MBW9266784.1 nitrogenase molybdenum-iron protein subunit beta *Candidatus* Thiodiazotropha sp. ex. *Lucinisca nassula*
MBT3010184.1 nitrogenase molybdenum-iron protein subunit beta *Candidatus* Thiodiazotropha sp. ex *Lucina pensylvanica*
MBT3094478.1 nitrogenase molybdenum-iron protein subunit beta *Candidatus* Thiodiazotropha sp. ex *Lucina pensylvanica*
MBT3014459.1 nitrogenase molybdenum-iron protein subunit beta *Candidatus* Thiodiazotropha taylori
MBT3029813.1 nitrogenase molybdenum-iron protein subunit beta *Candidatus* Thiodiazotropha sp. ex *Lucina pensylvanica*
WP 069124652.1 nitrogenase molybdenum-iron protein subunit beta *Candidatus* Thiodiazotropha endolucinida

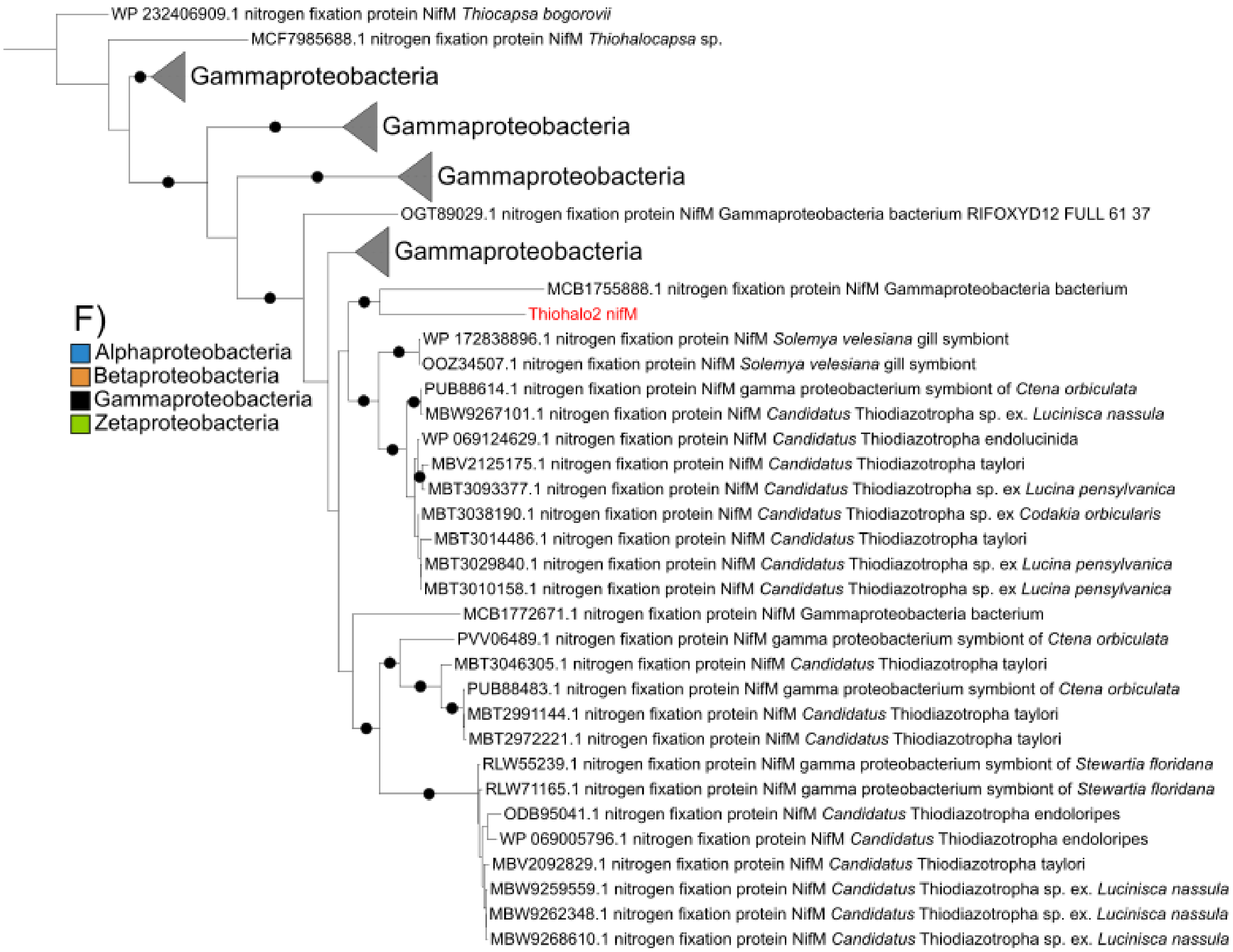

Figure S6: Phylogenetic relationships of Thiohalomonaldales nif genes to 50 best blast hits on NCBI. Shown is the NCBI phylogenetic tree reconstructed from the top hits from NCBI. Node colors indicate taxonomy of source sequences, with gray indicating that clade taxonomy is entirely Gammaproteobacteria. Trees are rooted artificially on the worst (50 of 50) blast hit. Black dots on branches indicate a Shimodaira-Hasegawa value of 0.9 or higher. A) *nifB* B) *nifD* C) *nifE* D) *nifH* E) *nifK* F) *nifM.* Alignments and original phylogenies are available on FigShare[16].

Figure S7

A)

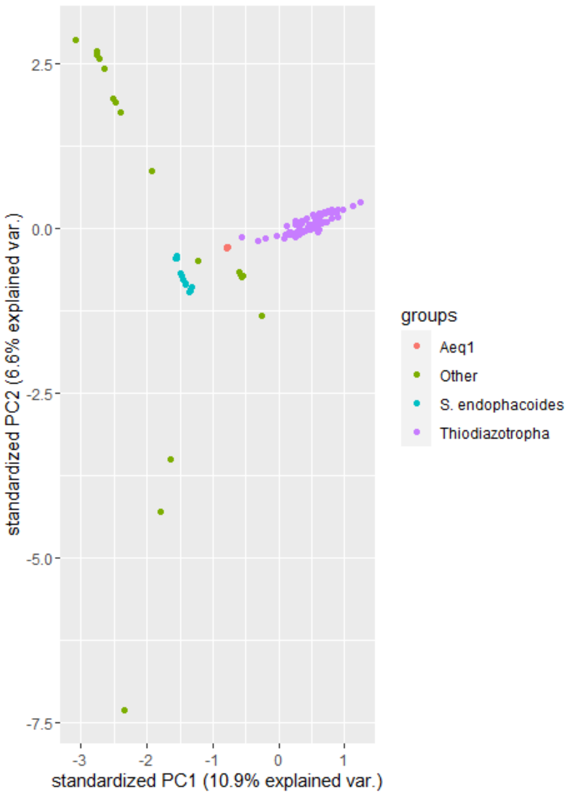
2.5
0.0
-2.5
-5.0
-7.5
-3
-2
-1
0
1
standardized PC1 (10.9% explained var.)
standardized PC2 (6.6% explained var.)
groups
Aeq1
Other
S. endophacoides
Thiodiazotropha

B)

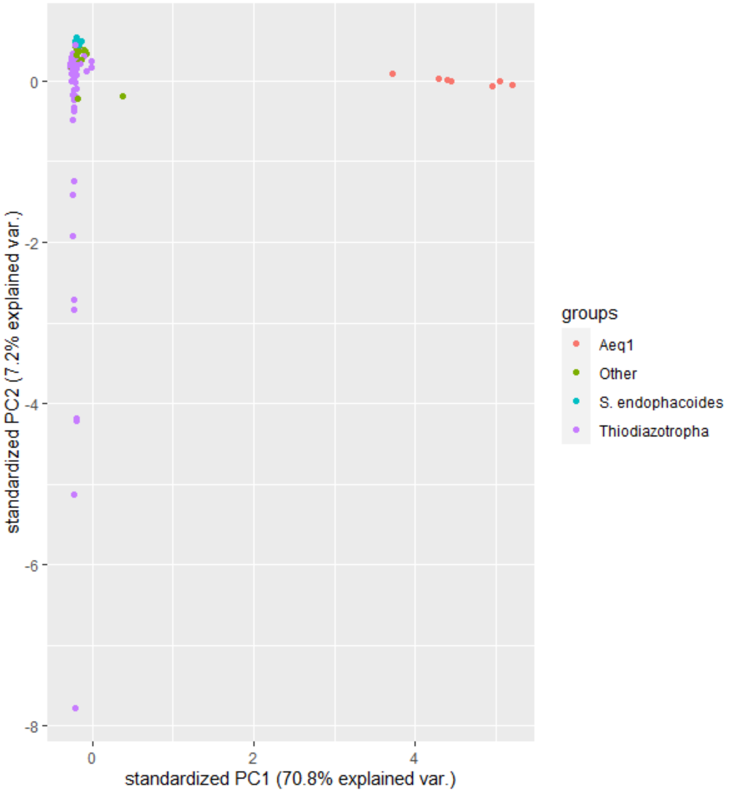

Figure S7: PCA of occurrence of eggNOG annotated functions of all MAGs used in study. A) Scaled PCA B) Unscaled PCA.

References

1 Mahram A, Herbordt MC. NCBI BLASTP on High-Performance Reconfigurable Computing Systems. *ACM Trans Reconfigurable Technol Syst* 2015; **7**: 1–20.

2 Katoh K, Standley DM. MAFFT multiple sequence alignment software version 7: improvements in performance and usability. *Mol Biol Evol* 2013; **30**: 772–780.

3 Price MN, Dehal PS, Arkin AP. FastTree 2--approximately maximum-likelihood trees for large alignments. *PLoS One* 2010; **5**: e9490.

4 Letunic I, Bork P. Interactive Tree Of Life (iTOL) v5: an online tool for phylogenetic tree display and annotation. *Nucleic Acids Res* 2021; **49**: W293–W296.

5 Glover EA, Taylor JD, Rowden AA. *Bathyaustriella thionipta*, a new lucinid bivalve from a hydrothermal vent on the Kermadec Ridge, New Zealand and its relationship to shallow-water taxa (Bivalvia: Lucinidae). *J Molluscan Stud* 2004; **70**: 283–295.

6 Lim SJ, Alexander L, Engel AS, Paterson AT, Anderson LC, Campbell BJ. Extensive Thioautotrophic Gill Endosymbiont Diversity within a Single *Ctena orbiculata* (Bivalvia: Lucinidae) Population and Implications for Defining Host-Symbiont Specificity and Species Recognition. *mSystems* 2019; **4**. doi:10.1128/mSystems.00280-19.

7 Osvatic JT, Wilkins LGE, Leibrecht L, Leray M, Zauner S, Polzin J *et al.* Global biogeography of chemosynthetic symbionts reveals both localized and globally distributed symbiont groups. *Proc Natl Acad Sci U S A* 2021; **118**. doi:10.1073/pnas.2104378118.

8 McCutcheon JP, Moran NA. Extreme genome reduction in symbiotic bacteria. *Nat Rev Microbiol* 2011; **10**: 13–26.

9 Taylor JD, Glover E. *Biology, Evolution and Generic Review of the Chemosymbiotic Bivalve Family Lucinidae*. Ray Society, 2021.

10 Hentschel U, Hand S, Felbeck H. The contribution of nitrate respiration to the energy budget of the symbiont-containing clam *Lucinoma aequizonata*: a calorimetric study. *J Exp Biol* 1996; **199**: 427–433.

11 Hentschel U, Cary SC, Felbeck H. Nitrate respiration in chemoautotrophic symbionts of the bivalve *Lucinoma aequizonata*. Marine Ecology Progress Series. 1993; **94**: 35–41.

12 Arndt-Sullivan C, Lechaire J-P, Felbeck H. Extreme Tolerance to Anoxia in the *Lucinoma aequizonata* Symbiosis. Journal of Shellfish Research. 2008; **27**: 119–127.

13 Hentschel U, Felbeck H. Nitrate Respiration in Chemoautotrophic Symbionts of the Bivalve *Lucinoma aequizonata* Is Not Regulated by Oxygen. *Appl Environ Microbiol* 1995; **61**: 1630–1633.

14 Lim SJ, Davis B, Gill D, Swetenburg J, Anderson LC, Engel AS *et al.* Gill microbiome structure and function in the chemosymbiotic coastal lucinid *Stewartia floridana*. *FEMS Microbiol Ecol* 2021; **97**. doi:10.1093/femsec/fiab042.

15 Duan Q, Zhou M, Zhu L, Zhu G. Flagella and bacterial pathogenicity. *J Basic Microbiol* 2013; **53**: 1–8.

16 Osvatic J. Supplementary Figure:nif gene BLAST results (raw phylogeny and alignments). 2022. doi:10.6084/m9.figshare.19335644.v1.
